# Socio-geographical disparities of obesity and excess weight in adults in Spain: insights from the ENE-COVID study

**DOI:** 10.3389/fpubh.2023.1195249

**Published:** 2023-07-17

**Authors:** Enrique Gutiérrez-González, Marta García-Solano, Roberto Pastor-Barriuso, Nerea Fernández de Larrea-Baz, Almudena Rollán-Gordo, Belén Peñalver-Argüeso, Isabel Peña-Rey, Marina Pollán, Beatriz Pérez-Gómez, the ENE-COVID Study Group

**Affiliations:** ^1^Spanish Agency for Food Safety and Nutrition, Madrid, Spain; ^2^Department of Epidemiology of Chronic Diseases, National Centre for Epidemiology, Institute of Health Carlos III, Madrid, Spain; ^3^Consortium for Biomedical Research in Epidemiology and Public Health (CIBERESP), Madrid, Spain

**Keywords:** obesity, adults, Spain, geographical factors, sociodemographic factors

## Abstract

**Background:**

In Spain, differences in the prevalence of obesity and excess weight according to sex and sociodemographic factors have been described at the national level, although current data do not allow to delve into geographical differences for these conditions. The aim was to estimate national and regional prevalences of adult obesity and excess weight in Spain by sex and sociodemographic characteristics, and to explore difference sources of inequalities in its distribution, as well as its geographical pattern.

**Method:**

ENE-COVID study was a nationwide representative seroepidemiological survey with 57,131 participants. Residents in 35,893 households were selected from municipal rolls using a two-stage random sampling stratified by province and municipality size (April–June 2020). Participants (77.0% of contacted individuals) answered a questionnaire which collected self-reported weight and height, as well as different socioeconomic variables, that allowed estimating crude and standardized prevalences of adult obesity and excess weight.

**Results:**

Crude prevalences of obesity and excess weight were higher in men (obesity: 19.3% vs. 18.0%; excess weight: 63.7% vs. 48.4%), while severe obesity was more prevalent in women (4.5% vs. 5.3%). These prevalences increased with age and disability, and decreased with education, census tract income and municipality size. Differences by educational level, relative census income, nationality or disability were clearly higher among women. Obesity by province ranged 13.3–27.4% in men and 11.4–28.1% in women; excess weight ranged 57.2–76.0% in men and 38.9–59.5% in women. The highest prevalences were located in the southern half of the country and some north-western provinces. Sociodemographic characteristics only explained a small part of the observed geographical variability (25.2% obesity).

**Conclusion:**

Obesity and overweight have a high prevalence in Spain, with notable geographical and sex differences. Socioeconomic inequalities are stronger among women. The observed geographical variability suggests the need to implement regional and local interventions to effectively address this public health problem.

## 1. Introduction

Overweight and obesity –or excess weight when both are considered-have become a major public health problem worldwide associated with high health and social costs, especially in the more developed countries. Excess weight could be responsible for 92 million premature deaths between 2020 and 2050 (1, 2) and its growing prevalence makes obesity prevention a priority in the near future (3–5). Recently, the World Health Organization (WHO) has estimated that 41% of males and 30% of females in the European Region have excess weight, while 22% of men and 24% of women have obesity (3). Excess weight increases the risk of developing many non-communicable diseases (NCDs) (e.g., cancer, cardiovascular diseases, type 2 diabetes mellitus, fatty liver, chronic respiratory diseases, or musculoskeletal disorders), impairs mental health and increases the risk of disability (3).

One of the main research areas in this field is the identification of inequalities related to excess weight and obesity distribution, including differences by sex, age, socioeconomic individual or contextual factors, or geographical patterns, as it may help to better understand these problems and to tailor public health interventions (4, 6). In Spain, the National Health Survey (ENSE) and the European Health Survey (EESE) monitor adult overweight and obesity prevalence, showing prevalences of excess weight in adult population of 54.5% (2017) and 53.6% (2020) respectively (7, 8). Although these surveys allow to estimate prevalences at the regional level, along with other regional surveys performed in some regions like Andalusia (excess weight 56.1% in 2015) (9) or Catalonia (excess weight 50.6% in 2020) (10), they do not allow to explore in detail the geographical variability of these indicators, and to date none of these studies has been performed with the appropriate design and sample size to estimate prevalences of obesity and excess at the provincial level.

In this context, the ENE-COVID seroepidemiological study, a large, longitudinal, nationwide representative population-based survey launched in 2020 to quantify prevalence of SARS-CoV-2 infection (11) provides a unique opportunity to carry out an in-depth analysis of geographical differences in excess weight. ENE-COVID, carried out by the Spanish Ministry of Health, the Instituto de Salud Carlos III and all the Spanish Regional Health Authorities, has more than 60,000 participants with self-reported anthropometric data and individual and contextual socioeconomic information (12). This is probably the largest representative population sample used to date to estimate the prevalence of obesity, with the added value of being specifically designed to provide representative estimates at the province level (Nomenclature of territorial units for statistics, NUTS 3).

Therefore, the aims of this work are: (a) to describe the prevalence of obesity and excess weight in adults in Spain, nationwide and by province, according to key sociodemographic characteristics; and (b) to evaluate gender, age, geographical and socioeconomic disparities in the prevalence of obesity and excess weight in Spain, and to estimate the contribution of these factors in the observed geographical patterns.

## 2. Materials and methods

### 2.1. Study design and participants

ENE-COVID is a nationwide population-based survey initially designed to estimate the prevalence of SARS-CoV-2 infection in the Spanish population during the first wave of the pandemic. The main features of ENE-COVID have been described elsewhere (11). Briefly, 1,500 census tracts were sampled with probability proportional to their size, stratifying by province and municipality size; then, up to 24 households were randomly selected within each census tract by the National Institute of Statistics (INE). Institutionalized people (care-home residents, hospitalized people, people in prisons, nuns and friars in convents, and residents in other collective institutions) were excluded from the study. All people residing in the 35,885 selected households were invited to participate, resulting in a final sample of 104,605 individuals of all ages. The first phase of the study design included three follow-up waves of data collection, with a 1-week break between them, conducted from April 27 to June 22, 2020. Field work was performed by staff from each of the region’s health departments following a common protocol developed by the Institute of Health Carlos III and the Ministry of Health. The selected households were contacted telephonically, and all residents were invited either to go to their primary health-care centres or to allow study nurses to visit their home. Participants answered an epidemiological questionnaire that included information about sociodemographic characteristics, self-reported anthropometric measurements, risk factors for severe COVID-19, disabilities and chronic conditions. The questionnaire and study protocol are available in Spanish in the ENE-COVID study website (12). We also obtained data on personal average income per each census tract from INE; in order to approximate it to the relative wealth of the census tract in each region, we sorted census tract by their income within each province and classified them by quartiles. All enrolled study participants signed a written informed consent (Supplementary material). The Institutional Review Committee of the Institute of Health Carlos III approved the study (register number PI 39_2020).

Of 104,600 selected individuals, 5,714 were not eligible, 10,238 could not be contacted and 20,361 refused to participate (Supplementary Figure S1). Of the remaining 68,287 study participants (65% response rate), for the current study we included data from participants aged 18 years or older that had correct weight and height data completed (*n* = 57,131: 47.3% men; 52.7% women).

### 2.2. Statistical analysis

Body mass index (BMI) of each participant was calculated as weight in kilograms divided by height in meters squared (13). For those individuals who participated in more than one of the three first waves of the study, we used mean height and weight for calculations. To define obesity and excess weight we used the following cut-offs, according to the WHO criteria: excess weight (BMI ≥25 kg/m^2^), obesity (BMI ≥30 kg/m^2^), and severe obesity (BMI ≥35 kg/m^2^). We calculated the prevalence of each weight status, as well as the population affected based on data from the latest census (14).

To control for possible confounding factors, the prevalence of each weight status by individual characteristics was standardized to the overall distribution of other sociodemographic characteristics in the entire Spanish population, including age, sex, nationality, education, relative average personal disposable income in the census section within the province, disability, and municipality size. For this purpose, we fitted a design-based logistic regression model adjusted for all these sociodemographic characteristics, and then computed a weighted average of the predicted probabilities of having each weight status, assuming that every participant was in each category of the individual characteristic (15). We estimated standardized prevalence ratios for each weight status across categories of each sociodemographic characteristic.

To account for the different sampling selection probabilities by province and to adjust for differential non-response based on sex, age, and average income in the census tracts, we assigned sampling weights to each study participant. Extreme weights (upper 0.5%) were trimmed to prevent highly influential observations. All statistical analyses took into account stratification by province and municipality size, and the clustering by household and census tract, when calculating standard errors (SEs) of prevalence estimates. Finite population corrections were applied since some sampling fractions of census tracts per stratum and households per census tract were not negligible. Confidence intervals (CIs) were calculated using logit-transformed prevalences and log-transformed ratios, with design-based degrees of freedom equal to the number of first-stage sampling units minus the number of strata, and they were back-transformed to the original scale for reporting.

We also quantified the proportion of the geographical variability in the prevalence of excess weight and obesity across the 52 Spanish provinces that was explained by differences in sociodemographic characteristics. Thus, we first calculated crude and standardized prevalences in each province using the same model-based standardization and adjustment factors described above. We then estimated the between-province variances in crude and standardized prevalences by fitting inverse-variance weighted random-effects meta-analyses on logit-transformed prevalences through the method of moments (16). The geographical variability in the prevalence of each weight status explained by sociodemographic characteristics was calculated as one minus the standardized-to-crude ratio of between-province variances.

Analyses were performed using survey commands in Stata (version 16).

## 3. Results

In a first approach, the crude prevalences of obesity (P_obes_) and excess weight (P_excess_) are shown in Table 1. Overall P_obes_ was 18.7% (95%CI: 18.1–19.2) while P_excess_ was 55.8% (95%CI: 55.1–56.4). In absolute numbers, that would correspond, approximately, to 8,762,000 people with obesity and 26,147,000 people with excess weight in the entire non-institutionalized Spanish population (12).

**Table 1:** Crude prevalence of obesity and excess weight in adult population by sociodemographic characteristics in ENE-COVID study.

	Total		Men		Women	
	*N*	% (95% CI)	*N*	% (95% CI)	*N*	% (95% CI)
Obesity (BMI ≥ 30 kg/m^2^)						
Overall	57,131	18.7 (18.1–19.2)	27,031	19.3 (18.7–20.0)	30,100	18.0 (17.4–18.7)
Nationality						
Spanish	54,559	18.7 (18.2–19.2)	25,947	19.4 (18.7–20.1)	28,612	18.0 (17.4–18.7)
Other	2,562	17.8 (16.1–19.7)	1,080	18.0 (15.5–20.7)	1,482	17.7 (15.4–20.2)
Education						
Less than primary	3,678	33.7 (31.7–35.7)	1,490	28.4 (25.6–31.5)	2,188	37.0 (34.5–39.6)
Primary	7,811	27.1 (25.8–28.4)	3,523	25.8 (24.0–27.7)	4,288	28.1 (26.4–29.9)
Secondary	31,164	18.6 (18.0–19.2)	15,674	19.8 (18.9–20.6)	15,490	17.3 (16.5–18.1)
University	12,587	10.8 (10.1–11.5)	5,352	12.9 (11.8–14.1)	7,235	9.0 (8.2–9.8)
Census tract average income*						
<25th percentile	15,229	22.4 (21.4–23.5)	7,230	21.8 (20.3–23.3)	7,999	23.1 (21.8–24.4)
25th–<50th percentile	14,397	20.3 (19.4–21.3)	6,838	21.6 (20.4–22.9)	7,559	19.1 (17.9–20.4)
50th–<75th percentile	13,054	17.5 (16.6–18.5)	6,221	17.9 (16.7–19.1)	6,833	17.2 (16.0–18.5)
≥75th percentile	14,451	14.2 (13.2–15.2)	6,742	15.8 (14.5–17.3)	7,709	12.7 (11.6–13.9)
Disability						
No	52,224	18.2 (17.7–18.7)	24,468	19.1 (18.4–19.8)	27,756	17.4 (16.7–18.1)
Yes	3,171	27.0 (25.1–28.9)	1,660	24.1 (21.7–26.7)	1,511	30.1 (27.3–33.0)
Municipality size (inhabitants)						
<5,000	10,401	22.2 (20.8–23.7)	5,160	23.4 (21.4–25.5)	5,241	21.0 (19.3–22.8)
5,000–19,999	12,045	20.1 (19.1–21.1)	5,730	20.6 (19.2–22.0)	6,315	19.6 (18.4–20.9)
20,000–99,999	17,006	18.4 (17.5–19.4)	7,939	19.4 (18.3–20.7)	9,067	17.5 (16.4–18.6)
≥100,000	17,679	17.2 (16.3–18.0)	8,202	17.5 (16.4–18.6)	9,477	16.9 (15.8–18.0)
Excess weight (BMI ≥ 25 kg/m^2^)						
Overall	57,131	55.8 (55.1–56.4)	27,031	63.7 (62.9–64.4)	30,100	48.4 (47.5–49.3)
Nationality						
Spanish	54,559	55.8 (55.1–56.5)	25,947	63.6 (62.9–64.4)	28,612	48.3 (47.4–49.3)
Other	2,562	55.8 (53.2–58.4)	1,080	64.2 (60.5–67.7)	1,482	49.1 (45.7–52.6)
Education						
Less than primary	3,678	75.3 (73.5–77.1)	1,490	75.8 (72.9–78.5)	2,188	75.0 (72.7–77.3)
Primary	7,811	70.7 (69.3–72.1)	3,523	75.1 (73.1–77.0)	4,288	67.2 (65.2–69.1)
Secondary	31,164	56.5 (55.7–57.2)	15,674	63.8 (62.8–64.7)	15,490	48.7 (47.7–49.7)
University	12,587	42.3 (41.1–43.4)	5,352	55.6 (53.8–57.3)	7,235	31.4 (30.0–32.9)
Census tract average income*						
<25th percentile	15,229	60.7 (59.6–61.8)	7,230	66.0 (64.5–67.4)	7,999	55.6 (54.1–57.2)
25th–<50th percentile	14,397	58.1 (56.8–59.4)	6,838	65.5 (63.9–67.1)	7,559	51.0 (49.3–52.7)
50th–<75th percentile	13,054	55.5 (54.2–56.8)	6,221	63.5 (61.9–65.0)	6,833	48.1 (46.3–49.9)
≥75th percentile	14,451	48.5 (47.1–49.9)	6,742	59.4 (57.8–61.0)	7,709	38.9 (37.1–40.7)
Disability						
No	52,224	55.3 (54.6–56.0)	24,468	63.4 (62.6–64.2)	27,756	47.7 (46.8–48.7)
Yes	3,171	66.9 (64.6–69.0)	1,660	69.9 (66.8–73.0)	1,511	63.5 (60.2–66.6)
Municipality size (inhabitants)						
<5,000	10,401	62.7 (61.2–64.2)	5,160	69.9 (68.2–71.5)	5,241	55.3 (53.2–57.4)
5,000–19,999	12,045	57.7 (56.3–59.0)	5,730	65.2 (63.4–66.8)	6,315	50.4 (48.6–52.1)
20,000–99,999	17,006	55.6 (54.4–56.8)	7,939	64.6 (63.2–66.0)	9,067	47.0 (45.5–48.6)
≥100,000	17,679	53.1 (51.9–54.3)	8,202	60.4 (59.1–61.7)	9,477	46.6 (45.0–48.2)

Population prevalence and 95% confidence intervals (CI) accounting for sampling weights, nonresponse rates by sex, age, and census tract average income, stratification by province and municipality size, and clustering by household and census tract. BMI, Body Mass Index; N, number of participants; *Categories based on percentiles from province-specific distributions of census tract average income in 2017.

**Figure 1:**
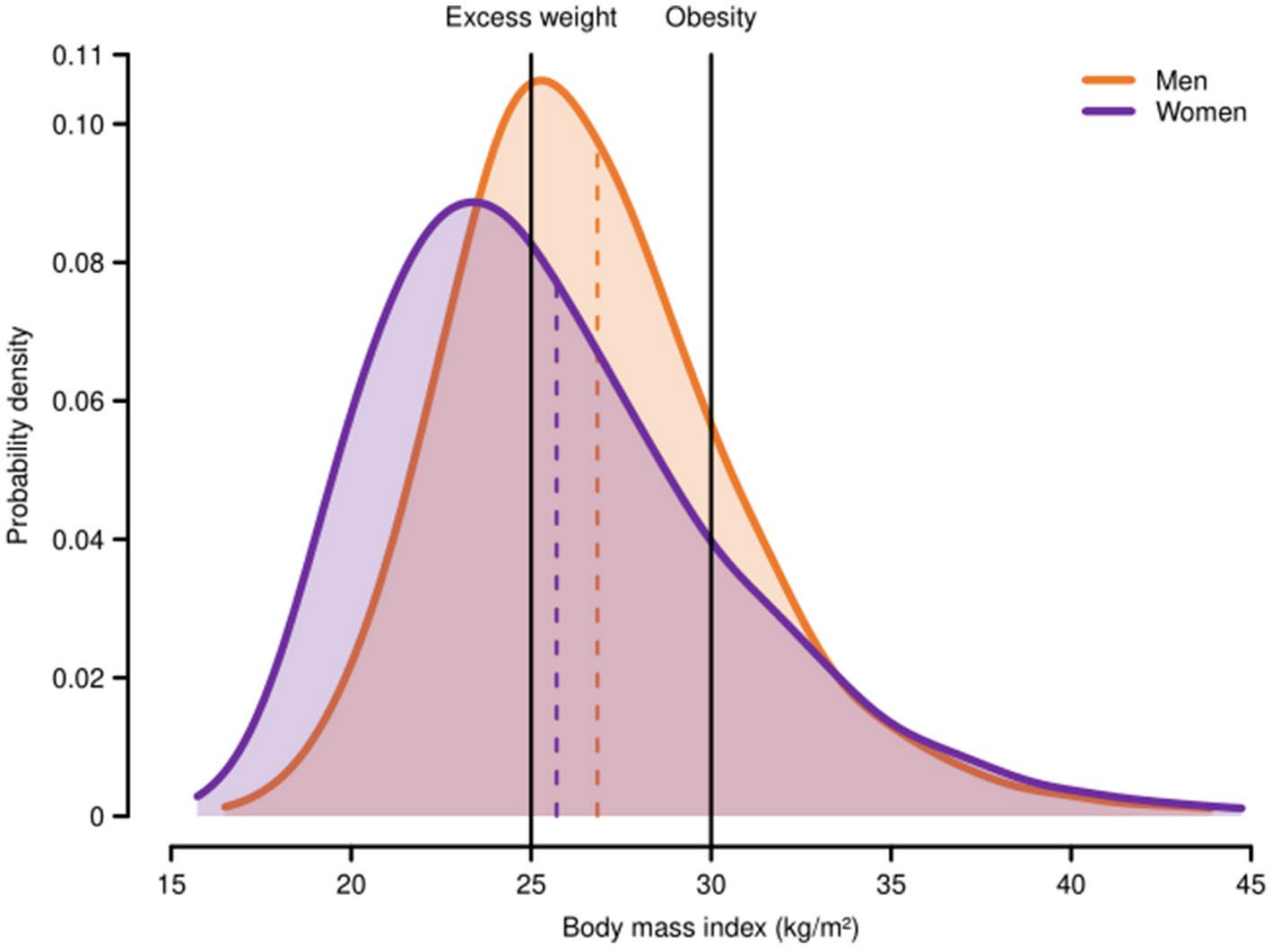
Body mass index distribution among adult men and women in Spain, ENE-COVID study, 2020. Probability density functions for body mass index among men and women were estimated using Gaussian kernel smoothers with a bandwidth of one-fifth their weighted standard deviations and applying sampling weights to account for the different selection probabilities and non-response rates of study participants. The vertical dashed lines represent the weighted means of body mass index among adult men and women (26.8 and 25.7 kg/m^2^, respectively). Excess weight (BMI ≥ 25 kg/m^2^); obesity (BMI ≥ 30 kg/m^2^).

Both conditions, especially excess weight, were more prevalent in men. As for severe obesity (BMI > 40 kg/m^2^), the prevalence was 4.9% (95%CI: 4.6–5.1), being slightly higher in women (5.3; 95%CI: 5.0–5.6) (Supplementary Table S1). As shown in Figure 1, BMI distribution differed by sex: mean BMI was higher in men than in women (26.8 kg/m^2^; 95%CI: 26.8–26.9 vs. 25.7 kg/m^2^; 95%CI: 25.6–25.8, respectively) while interquartile range (IQR) was wider in women (5.2 kg/m^2^ vs. 6.4 kg/m^2^).

**Figure 2:**
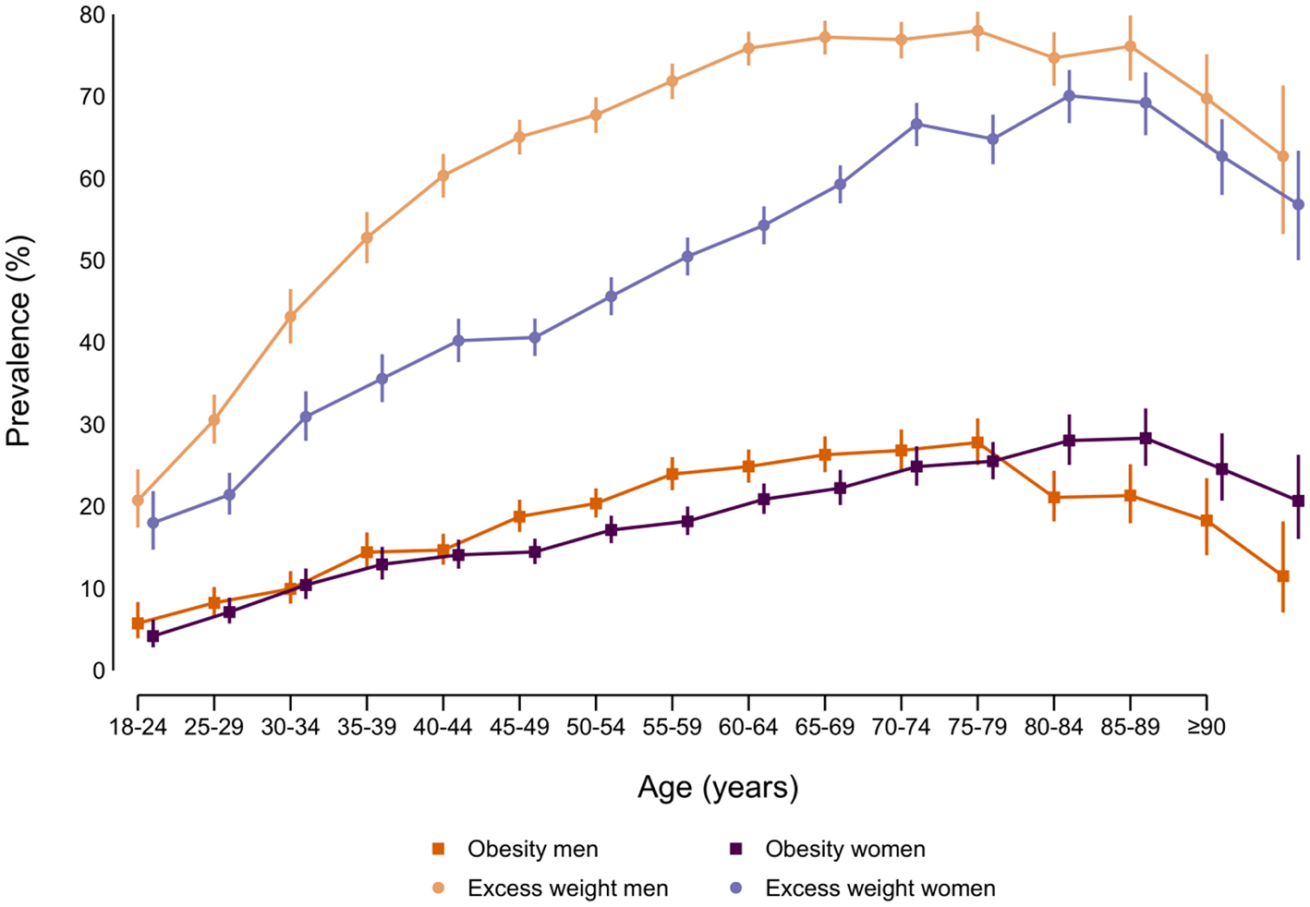
Prevalence (%) of obesity and excess weight by sex and age group in adult participants from ENE-COVID study.

When we combined sex and age, we observed that in both, men and women, the prevalence of obesity and excess weight increased with age until the eldest groups, in which both prevalences declined (Figure 2; Supplementary Table S2); however, there were marked differences depending on which indicator we looked at: P_excess_ was clearly higher in men across all age groups, especially in middle ages (25–65 years). In contrast, prevalence of obesity in men was similar or slightly higher than in women until age of 65–69 years, after which it decreased and was lower among men. Of note, for severe obesity, the prevalence in women was higher than in men since middle age (50–55 years) (Supplementary Table S1).

As for the distribution of the two indicators of adiposity according to socioeconomic factors (crude prevalences, Table 1), although we observed an inverse gradient in both sexes with the level of education attained and with the relative average personal disposable income in the census section within the province, the differences were clearly more pronounced in women, especially for obesity. Also, participants with recognized disability (>33%) had higher prevalences in the adiposity estimators than those with no disability, especially among women. There were no differences among Spaniards and those with other nationalities, while, for municipality size, the highest prevalences were found in rural areas (municipality size <5,000 inhabitants). Gradients in the same direction than the ones seen for P_obes_ and P_excess_ were found for severe obesity (Supplementary Table S1).

**Figure 3:**
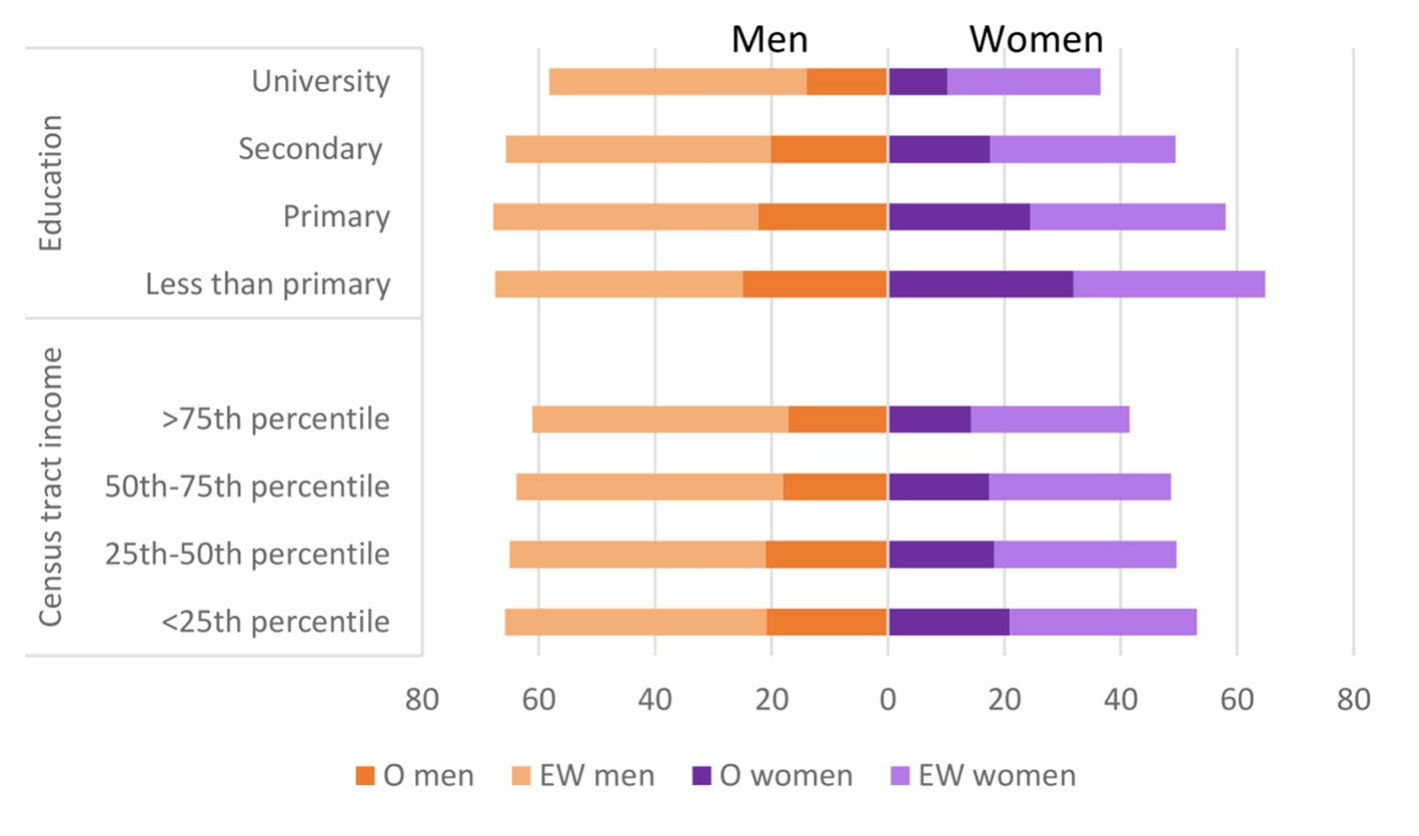
Standardized* prevalences (%) of obesity and excess weight by level of education, census tract income and sex in adult participants in ENE-COVID study. *Standardized to the overall distribution of age, nationality, education, census tract income, disability and municipality size. O, obesity; EW, excess weight.

In a second step, we calculated the prevalence of excess weight and of obesity standardized to the overall distribution of the sociodemographic variables in the study population (namely, age, sex, nationality, education, disability, census tract income and municipality size), as well as the ratios of these standardized prevalences across the categories of each variable (Table 2 for obesity and Table 3 for excess weight). Our results show that, even after standardization, P_obes_ increased with age, was higher among men and among those with disability, as well as among those residing in those census tracts with the lowest average personal disposable income within each province. Similar results were observed for standardized P_excess_, although in this case, it was also related to foreign nationality and to smaller municipality size. It is interesting to note that, even though standardized P_obes_ and P_excess_ were higher in men, the inverse gradient between these indicators with level of education or with relative census tract income was clearly more evident in women (Figure 3), as were the increased prevalences in those with disability or of non-Spanish nationality.

**Table 2:** Standardized^*^ prevalence and prevalence ratio of obesity by sociodemographic variables in ENE-COVID study.

	Total (*N* = 57,131)		Men (*N* = 27,031)		Women (*N* = 30,100)	
	Standardized prevalence	Standardized prevalence ratio	Standardized prevalence	Standardized prevalence ratio	Standardized prevalence	Standardized prevalence ratio
	% (95% CI)	Ratio (95% CI)	% (95% CI)	Ratio (95% CI)	% (95% CI)	Ratio (95% CI)
Age group (years)						
18–29	8.9 (8.1–9.8)	Ref.	8.6 (7.5–9.9)	Ref.	9.3 (8.2–10.6)	Ref.
30–39	15.3 (14.2–16.5)	1.72 (1.53–1.95)	14.7 (13.3–16.2)	1.70 (1.43–2.03)	16.2 (14.8–17.8)	1.75 (1.49–2.05)
40–49	18.9 (18.0–19.9)	2.13 (1.92–2.37)	20.0 (18.7–21.4)	2.32 (1.99–2.70)	17.9 (16.6–19.4)	1.93 (1.66–2.24)
50–59	22.2 (21.2–23.3)	2.50 (2.25–2.78)	24.3 (22.9–25.9)	2.82 (2.42–3.28)	20.3 (18.8–21.8)	2.18 (1.90–2.50)
60–69	23.8 (22.7–25.0)	2.68 (2.40–2.99)	25.8 (24.1–27.4)	2.98 (2.56–3.48)	21.8 (20.2–23.5)	2.34 (2.02–2.72)
70–79	22.4 (21.0–23.9)	2.52 (2.25–2.83)	23.3 (21.3–25.5)	2.70 (2.30–3.17)	21.1 (19.1–23.3)	2.27 (1.94–2.66)
≥80	17.8 (16.2–19.6)	2.00 (1.74–2.30)	16.4 (14.1–18.9)	1.89 (1.54–2.33)	17.6 (15.3–20.1)	1.89 (1.57–2.27)
Sex						
Men	19.4 (18.8–20.1)	1.09 (1.04–1.13)				
Women	17.9 (17.3–18.6)	Ref.				
Nationality						
Spanish	18.6 (18.1–19.1)	Ref.	19.4 (18.7–20.1)	Ref.	17.8 (17.1–18.5)	Ref.
Other	19.6 (17.6–21.6)	1.05 (0.94–1.17)	19.2 (16.5–22.2)	0.99 (0.85–1.16)	19.5 (16.8–22.6)	1.10 (0.96–1.26)
Education						
Less than primary	29.2 (27.2–31.3)	2.42 (2.18–2.67)	25.0 (22.3–27.9)	1.79 (1.55–2.07)	31.9 (29.2–34.8)	3.09 (2.70–3.54)
Primary	23.4 (22.1–24.6)	1.93 (1.77–2.11)	22.3 (20.6–24.0)	1.60 (1.41–1.80)	24.4 (22.8–26.2)	2.37 (2.10–2.67)
Secondary	18.8 (18.2–19.5)	1.56 (1.45–1.67)	20.1 (19.3–21.0)	1.44 (1.31–1.59)	17.5 (16.6–18.4)	1.70 (1.54–1.87)
University	12.1 (11.3–12.9)	Ref.	14.0 (12.8–15.3)	Ref.	10.3 (9.1–11.6)	Ref.
Census tract average income (percentile)						
<25th	21.0 (20.0–22.1)	1.33 (1.23–1.45)	20.9 (19.6–22.4)	1.22 (1.09–1.36)	20.9 (19.6–22.3)	1.46 (1.31–1.63)
25th–<50th	19.6 (18.7–20.6)	1.25 (1.15–1.35)	21.0 (19.8–22.2)	1.22 (1.10–1.36)	18.2 (17.1–19.5)	1.28 (1.14–1.42)
50th–<75th	17.8 (16.9–18.7)	1.13 (1.04–1.22)	18.0 (16.8–19.3)	1.05 (0.94–1.17)	17.4 (16.2–18.7)	1.22 (1.09–1.36)
≥75th	15.8 (14.8–16.8)	Ref.	17.2 (15.8–18.6)	Ref.	14.3 (12.9–15.8)	Ref.
Disability						
No	18.4 (17.9–19.0)	Ref.	19.3 (18.6–20.0)	Ref.	17.4 (16.8–18.1)	Ref.
Yes	22.0 (20.5–23.6)	1.19 (1.11–1.29)	20.2 (18.1–22.4)	1.05 (0.94–1.17)	24.1 (22.0–26.3)	1.38 (1.25–1.53)
Municipality size (inhabitants)						
<5,000	19.9 (18.5–21.3)	1.08 (1.00–1.18)	21.2 (19.4–23.2)	1.15 (1.03–1.28)	18.5 (16.7–20.4)	1.03 (0.93–1.15)
5,000–19,999	19.2 (18.3–20.1)	1.05 (0.99–1.12)	19.7 (18.3–21.1)	1.06 (0.97–1.17)	18.7 (17.3–20.1)	1.04 (0.96–1.14)
20,000–99,999	18.3 (17.4–19.2)	1.00 (0.94–1.06)	19.4 (18.3–20.6)	1.05 (0.97–1.14)	17.0 (15.9–18.2)	0.95 (0.88–1.03)
≥100,000	18.3 (17.5–19.1)	Ref.	18.5 (17.4–19.6)	Ref.	17.9 (16.8–19.0)	Ref.

^*^Standardized to the overall distribution of age, sex, nationality, education, census tract income, disability and municipality size.

**Table 3:** Standardized^*^ prevalence and prevalence ratio of excess weight by sociodemographic variables in ENE-COVID study.

	Total (*N* = 57,131)		Men (*N* = 27,031)		Women (*N* = 30,100)	
	Standardized prevalence	Standardized prevalence ratio	Standardized prevalence	Standardized prevalence ratio	Standardized prevalence	Standardized prevalence ratio
	% (95% CI)	Ratio (95% CI)	% (95% CI)	Ratio (95% CI)	% (95% CI)	Ratio (95% CI)
Age group (years)						
18–29	30.2 (28.7–31.8)	Ref.	33.8 (31.8–36.0)	Ref.	27.1 (25.1–29.3)	Ref.
30–39	49.0 (47.5–50.5)	1.62 (1.53–1.72)	56.8 (54.7–58.9)	1.68 (1.56–1.81)	41.9 (39.8–44.0)	1.55 (1.42–1.68)
40–49	56.1 (55.0–57.3)	1.86 (1.77–1.96)	66.6 (65.0–68.1)	1.97 (1.84–2.10)	46.4 (44.9–48.0)	1.71 (1.58–1.85)
50–59	63.1 (61.9–64.3)	2.09 (1.98–2.20)	73.7 (72.1–75.1)	2.18 (2.04–2.32)	53.2 (51.7–54.7)	1.96 (1.82–2.12)
60–69	69.0 (67.7–70.2)	2.29 (2.17–2.41)	76.9 (75.4–78.4)	2.27 (2.13–2.43)	61.3 (59.8–62.9)	2.26 (2.09–2.44)
70–79	68.7 (66.9–70.4)	2.27 (2.15–2.41)	75.7 (73.5–77.8)	2.24 (2.09–2.40)	61.4 (59.3–63.5)	2.26 (2.08–2.46)
≥80	63.2 (60.8–65.5)	2.09 (1.96–2.24)	70.4 (67.0–73.7)	2.08 (1.92–2.26)	55.0 (51.7–58.4)	2.03 (1.84–2.24)
Sex						
Men	64.1 (63.3–64.8)	1.33 (1.30–1.35)				
Women	48.3 (47.3–49.2)	Ref.				
Nationality						
Spanish	55.7 (55.0–56.3)	Ref.	63.8 (63.0–64.6)	Ref.	47.9 (47.2–48.7)	Ref.
Other	61.0 (58.6–63.4)	1.10 (1.05–1.14)	67.8 (64.2–71.2)	1.06 (1.01–1.12)	54.3 (50.8–57.8)	1.13 (1.07–1.20)
Education						
Less than primary	67.1 (64.9–69.2)	1.43 (1.37–1.49)	67.5 (64.2–70.6)	1.16 (1.10–1.23)	64.8 (61.6–68.0)	1.77 (1.66–1.90)
Primary	62.9 (61.3–64.4)	1.34 (1.29–1.39)	67.8 (65.6–70.0)	1.16 (1.11–1.22)	58.0 (55.8–60.2)	1.59 (1.50–1.68)
Secondary	57.2 (56.5–58.0)	1.22 (1.18–1.25)	65.6 (64.6–66.5)	1.13 (1.09–1.16)	49.4 (48.5–50.4)	1.35 (1.29–1.42)
University	47.0 (45.8–48.2)	Ref.	58.2 (56.6–59.9)	Ref.	36.5 (34.9–38.2)	Ref.
Census tract average income (percentile)						
<25th	59.2 (58.1–60.3)	1.15 (1.12–1.19)	65.6 (64.2–67.0)	1.07 (1.03–1.10)	53.1 (51.7–54.6)	1.28 (1.22–1.35)
25th–<50th	57.1 (55.9–58.4)	1.11 (1.08–1.15)	65.0 (63.5–66.5)	1.06 (1.02–1.09)	49.6 (48.1–51.1)	1.20 (1.13–1.26)
50th–<75th	56.1 (54.9–57.3)	1.09 (1.06–1.13)	64.0 (62.4–65.5)	1.04 (1.00–1.08)	48.6 (47.1–50.2)	1.17 (1.11–1.23)
≥75th	51.3 (50.0–52.5)	Ref.	61.6 (60.0–63.1)	Ref.	41.5 (40.0–43.0)	Ref.
Disability						
No	55.8 (55.1–56.5)	Ref.	64.1 (63.3–64.9)	Ref.	47.9 (47.1–48.7)	Ref.
Yes	57.9 (55.6–60.1)	1.04 (1.00–1.08)	62.7 (59.5–65.8)	0.98 (0.93–1.03)	54.1 (50.9–57.2)	1.13 (1.06–1.20)
Municipality size (inhabitants)						
<5,000	58.9 (57.3–60.5)	1.07 (1.04–1.11)	67.5 (65.7–69.2)	1.09 (1.05–1.13)	50.9 (49.1–52.6)	1.06 (1.00–1.11)
5,000–19,999	56.5 (55.3–57.8)	1.03 (1.00–1.06)	64.7 (63.0–66.4)	1.04 (1.01–1.08)	48.9 (47.2–50.6)	1.01 (0.97–1.06)
20,000–99,999	55.8 (54.7–56.9)	1.02 (0.99–1.04)	65.2 (63.9–66.5)	1.05 (1.02–1.08)	46.9 (45.6–48.2)	0.97 (0.94–1.01)
≥100,000	54.9 (53.9–56.0)	Ref.	62.0 (60.7–63.3)	Ref.	48.2 (46.9–49.5)	Ref.

^*^Standardized to the overall distribution of age, sex, nationality, education, census tract income, disability and municipality size.

**Figure 4:**
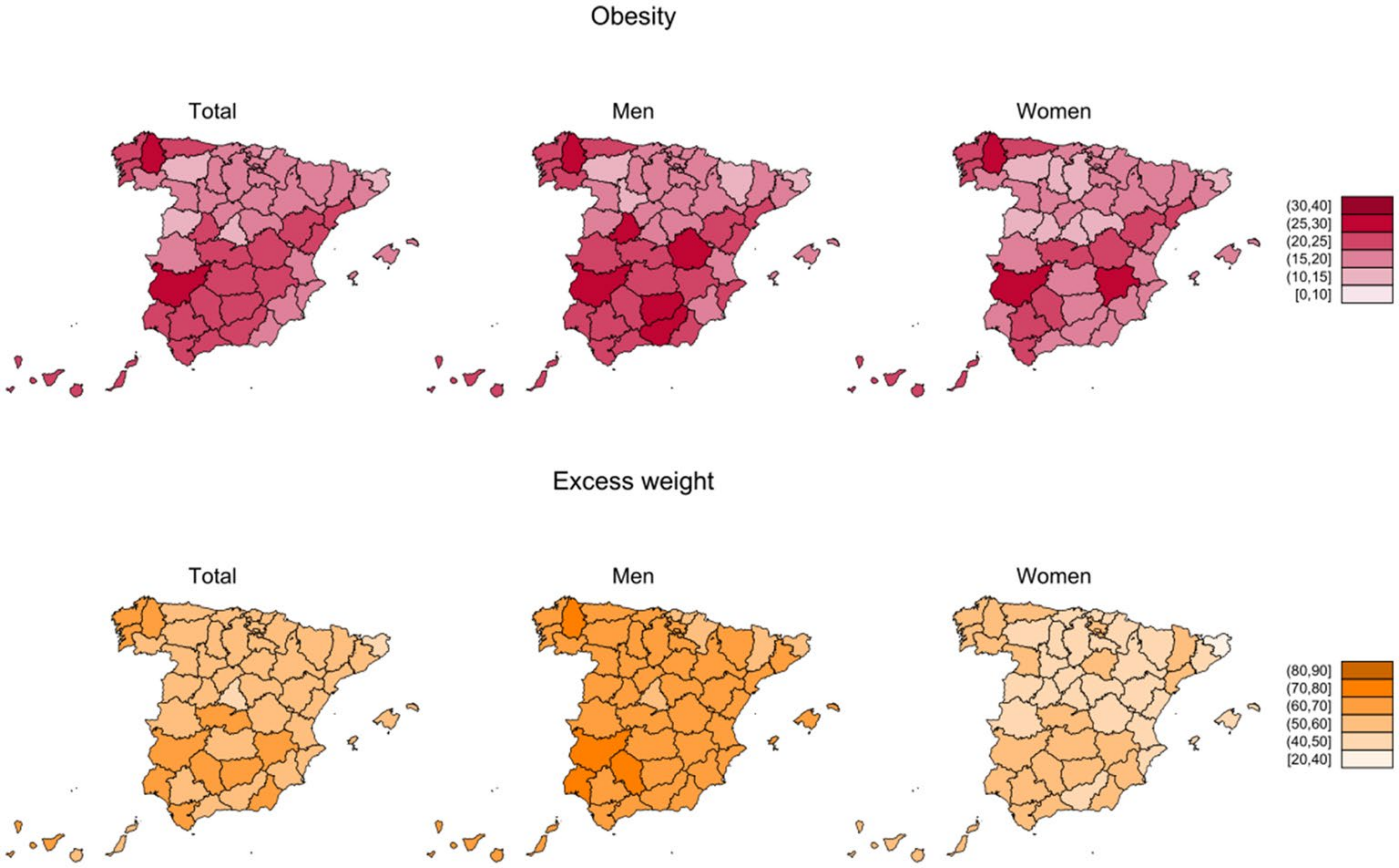
Crude prevalences (%) of obesity and excess weight by province and sex in adult participants from ENE-COVID study.

Third, we also explored the geographical distribution of crude P_obes_ and P_excess_ (Figure 4) overall and by sex (numerical figures are provided in Supplementary Tables S3, S4, and the name and location of each province is described in Supplementary Figure S2). For obesity, provincial prevalences ranged between 13.3 and 27.4% in men and between 11.4 and 28.1% in women. The highest P_obes_ in both sexes were found, in general, in those regions in the southern half of the country, and in some provinces in the north-western corner of Spain. The provinces with the highest P_obes_ were Badajoz (27.4% men; 26.7% women) and Lugo (26.5% men; 28.1% women). For excess weight, provincial prevalence ranged between 57.2 and 76.0% in men and between 38.9 and 59.5% in women. The geographic pattern of P_excess_ was quite similar to that of P_obes_, although less marked. In men, the highest P_excess_ were observed in Lugo (76.0%) and Córdoba (72.7%) and in women in Badajoz (59.5%) and Lugo (58.5%).

**Figure 5:**
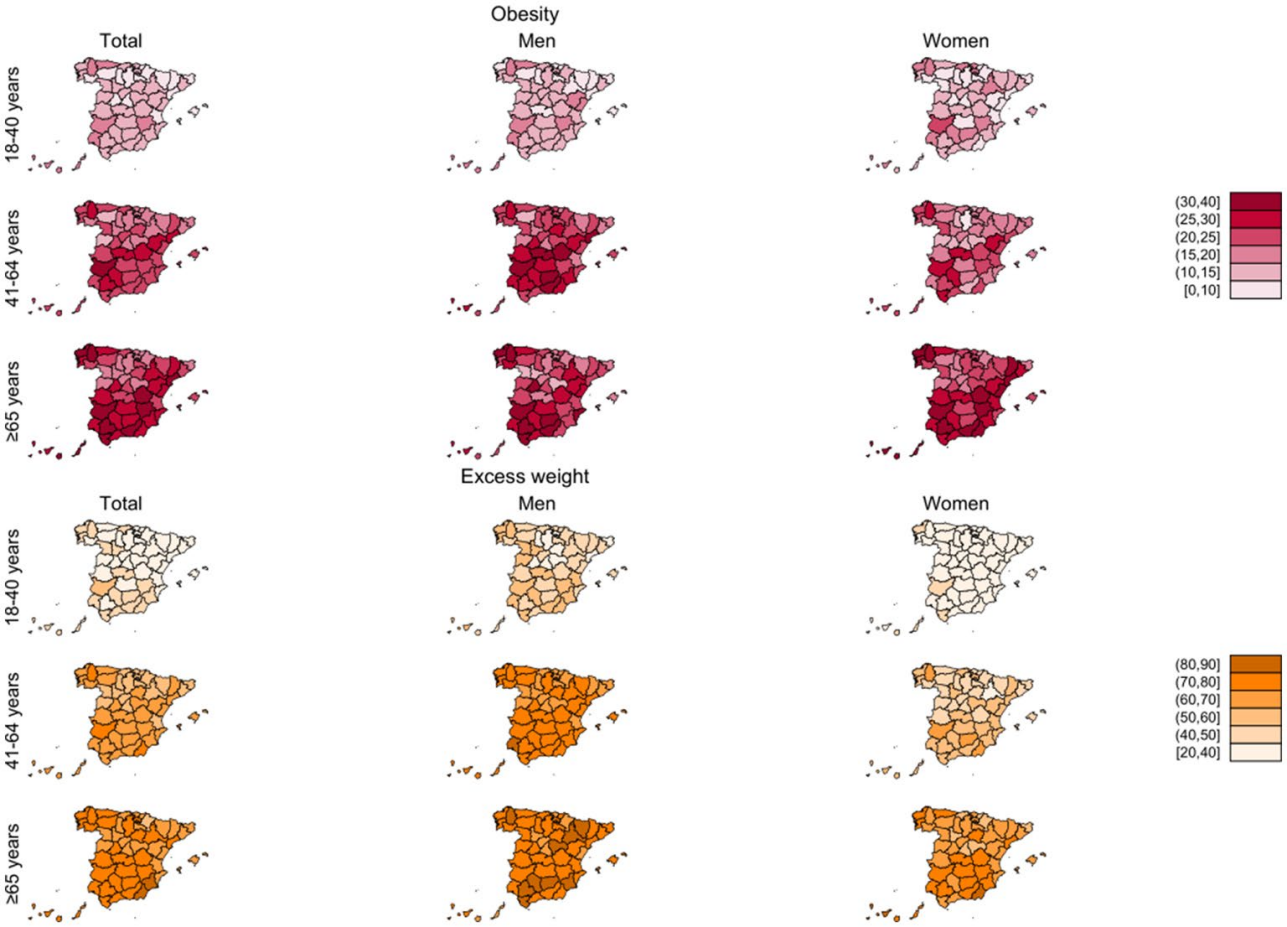
Crude prevalences of obesity and excess weight (%) by province, sex and age group in adult participants from ENE-COVID study.

Figure 5 represents P_obes_ and P_excess_ by province, age group and sex. In general, men had higher prevalences than women across all age groups. Of note, at older ages, prevalences of excess weight were over 70% in many provinces, especially among men, and, in several provinces, prevalence of obesity was over 30%, mainly in the group of 65 years and older. Age-standardized P_obes_ and P_excess_ geographical patterns by sex were similar to those of crude prevalences (Supplementary Figure S3; Supplementary Tables S5, S6).

Finally, we estimated the proportion of the geographical variability in the prevalence of excess weight and obesity that could be explained by the selected sociodemographic characteristics, including age, sex, nationality, education, census tract income, disability, and municipality size. According to our models, these variables explained only 25.2% of the variability in crude prevalences of obesity across provinces (24.0% in men and 22.0% in women).

## 4. Discussion

In this report, with data from the ENE-COVID study, we have been able to provide representative estimates of obesity and excess weight prevalence in adults in Spain, at both national and provincial levels. Our results highlight the magnitude of this public health problem in the country, with nearly 9 million people suffering (18.7%) from obesity and more than 26 million (55.8%) having excess weight. On the other hand, although men have higher prevalences of obesity and excess weight, inequalities in prevalence figures in relation to socioeconomic factors are more evident among women. Also of interest is the high geographical variability observed. According to our approach, only 25% of the differences in the prevalence of obesity between provinces can be explained by basic structural characteristics that are difficult to modify in the short term, such as population structure by age and sex or socioeconomic characteristics of the subjects.

The prevalences of obesity and excess weight in our study are slightly higher than those found by the last European Health Survey in Spain (8), also based on self-reported data from July 2019 to July 2020 (16.0 and 53.6%, respectively). The special context of ENE-COVID, with interviews conducted by healthcare personnel within a study that provided participants with clinical outcomes at a time of great health concern (i.e., presence of SARS-CoV-2 IgG in the first wave of the pandemic), may have contributed to better self-reporting of weight and height. Another possible explanation might be a certain increase in the prevalences as a consequence of the strong national lockdown enforced during this first epidemic wave. Nevertheless, both surveys show that more than half of the Spanish population have excess weight. These figures, although alarming, are below the estimates for the WHO European region average (P_obes_ 23%; P_excess_ 59%) (3).

Consistent with previous studies (8, 17), the prevalence of both conditions was higher in men, although the difference was more noticeable in the case of excess weight. The higher prevalences among men did not change after standardization by sociodemographic factors, pointing to the influence of other determinants. These results are in line with the estimates reported for almost all countries within the European Union for 2016. In contrast, in the rest of the countries of the WHO European region, obesity is more prevalent among women (3). Perhaps the higher caloric intakes could partially explain these differences (18). In addition, men tend to exercise more than women and, in general, have greater muscle mass, which would also result in a higher BMI. This is precisely one of the limitations of BMI, as it does not differentiate between the components of fat and lean body mass (19).

A noteworthy aspect is the increase in obesity and especially in excess weight with age, from the beginning of adulthood, although with differences between men and women, more evident for excess weight: prevalence increases more rapidly at early ages in men followed by a later stabilization, while in women it increases progressively until old age. It is well known that physical activity usually decreases with age, while energy intake remains more stable, which may partially contribute to higher adiposity in middle-aged and older adults (20, 21). On the other hand, the decrease of prevalence in older adults may be explained by different factors, including survival bias, as people with obesity have an increased mortality risk and a lower life expectancy (22), as well as the loss of lean body mass associated with aging (19). Some studies have also reported that age clearly influences food habits, showing, for instance, a higher consumption of fruits, vegetables or fish among people aged 65–75 years compared with younger participants, which may also explain the slight decrease in excess weight at this age (23).

Another relevant focus of attention in the epidemiology of excess weight/obesity are inequalities in their prevalence according to socioeconomic variables. In this case, we have used two complementary indicators: level of attained education and relative average personal disposable income in the census section within the province, a contextual indicator of the characteristics of the area of residence. As we have shown, despite higher prevalences of obesity and excess weight in men, the greater socioeconomic inequalities were seen among women. This is also true for other factors like disability and nationality (higher prevalences in non-Spanish participants), as described in previous studies (24, 25). We also observed that the differences between men and women by education were greater than those observed by census tract income level. Lower level of education has been associated with worst estimators in physical activity (17), especially among women (26), as well as worse eating habits, such as lower consumption of fruits and vegetables (27) or adherence to the Mediterranean diet (17). However, there are other gender-related reasons that can help to understand differences in social inequalities among men and women. Women with lower socioeconomic status continue to play a different role compared to their female peers with higher socioeconomic status, and have fewer opportunities for leisure-time physical activity, displaying more sedentary behavior, while these role differences by socioeconomic status are not as marked among men (26, 28).

One of the novelties of our study is that we provide for the first time in Spain representative figures of prevalence of obesity and excess weight among people with disabilities. They showed higher prevalences of both conditions, although after standardization this excess was seen among females. Sedentary lifestyle and lower degree of physical activity may in part explain these differences (29–31), as well as the adherence to an unbalanced diet (30). However, the relationship could also be inverse, since obesity and excess weight are also risk factors for disability (32). We also explored differences according to the urban/rural gradient: our results showed that obesity and excess weight were also more frequent in rural areas compared to large cities, an aspect already described in previous studies (33). However, these differences were attenuated or disappeared after standardizing for sociodemographic variables. Probably, the urban/rural gradient is explained by differences in age (higher among the rural population), and educational level (lower in rural areas).

The other relevant information provided by our study is the geographical pattern of the two adiposity indicators. For the first time, we provide representative estimates of the prevalence of obesity and excess weight at provincial level for the whole country, globally, by sex and by age groups. As our results show, there are remarkable geographical differences among Spanish provinces, with higher prevalences in the south and the north-western corner of Spain. Our results are in line with published estimates in studies on specific provinces (8, 17, 34). Also, our estimates are similar to those from single-province regions like Madrid (prevalence of excess weight 48.4% in 2020) (35) or Asturias (prevalence of excess weight 54.0% in 2017) (36). We tried to quantify how much of these differences could be explained by the disparities in structural characteristics among provinces that may be relevant in this context (37, 38) such as age and sex distribution, level of education, prevalence of disability, immigration, relative socioeconomic level and types of municipalities included. For instance, most of the provinces with the highest prevalence of obesity are also those with the highest number of low average-income towns (39). However, the age-standardized prevalences and the standardized prevalences across categories of sociodemographic variables still showed notable disparities, and our model indicated that these structural variables accounted roughly for a 25% of the geographical variability. Thus, a big part of this heterogeneity remained unexplained by these factors, which adds evidence for the potential effect of other determinants (genetic, cultural, dietary, physical activity, environmental…) that may be associated to differences between provinces (40, 41). Key factors that could be responsible for part of these differences may be related to lifestyle and habits. For instance, there are well known geographical disparities in food choices, energy and nutrient intake across Spain (42–44). In fact, despite the lower adherence to the Mediterranean dietary pattern observed in younger generations, Spanish population still maintains a high dietary diversity (45). Additionally, some characteristics like access to green spaces have been associated to BMI and obesity in Spain (38). Also, a positive relationship has been reported in Spain between ambient temperature and the prevalence of obesity, that remained after adjustment for sociodemographic and lifestyle factors, and that could be explained by the role of brown adipose tissue (46), and it is consistent with our findings for southern provinces. Additionally, other factors like employment rates or different social norms and standards regarding body shape, could be responsible for another part of the unexplained variability among provinces (40).

Our results have shown relevant geographical differences in the prevalence of obesity and excess weight in Spain, highlighting the need to deepen in the study and surveillance of obesity not only at the national and regional level, but also at the provincial and local level. This heterogeneous distribution of obesity prevalence has direct implications for policy development, and especially in Spain, since many public health policies are decided at regional, provincial or municipal level. Most of the policies or strategies to combat obesity have been designed at the national level, and are specially focused on child and adolescent population (47). For this reason, it is important to design new interventions (i.e., nutrition education programs at the local level, creation of sport facilities, local barriers to obesogenic environment, taxes, etc.), taking into account the prevalence at the local level, especially in territories with worrying obesity figures and widening their scope to the entire population.

One of the main strengths of our study is its large sample size (more than twice the National Health Survey’s) and its design, that allows to estimate accurate representative provincial prevalences of obesity and excess weight. Thus, we have been able to appreciate the geographical distribution of these conditions and to explore their association with key sociodemographic determinants. In addition, the high rate of participation as well as the inclusion of postestimation weights to take into account non-response differences, contribute to the reliability and validity of our results. In Europe, surveillance systems including obesity or overweight are only available for some regions, precluding comparisons not only at provincial level but also between countries (48–50). We have also been able to explore different sources of inequality, and provide a global picture of obesity and excess of weight in Spain.

On the other hand, perhaps the most noteworthy limitation of our study is the use of self-reported anthropometric data, which tends to underestimate the real BMI compared to objectively measured weight and height, with differences in the obesity prevalence between self-reported and objectively measured data ranging from 1 to 10 percentage points (51). Another limitation is the exclusion of institutionalized population, although its members represent only a small percentage of the population residing in Spain (<0.95% according to the last census) (14). In addition, the epidemiological questionnaire, which was mainly focused in COVID-19 risk factors, lacked information on key variables such as dietary habits, or previous physical activity, so it was not possible to evaluate their contribution in the observed geographical patterns. Finally, given the cross-sectional design of our analyses, we cannot establish causality in the associations observed between adiposity indicators and some of the variables studied.

## 5. Conclusion

More than half of the adult population in Spain has excess weight and almost one out of five residents in the country has obesity. Excess weight and obesity prevalences are higher with increasing age, in men, and, among women, in those with non-Spanish nationality or with any disability. Also, lower educational level, rurality and living in census tracts with lower relative income are associated to higher prevalences of the two adiposity indicators. Sociodemographic inequalities were greater among women. The prevalences of excess weight and obesity were very different among the Spanish provinces, with higher figures in the south and the north-western corner of Spain. Sociodemographic factors only explained a small proportion of the geographic variability. Therefore, there is a need to identify other potentially modifiable factors, and target them in national regional and local public health interventions.

## Data availability statement

The datasets presented in this article are not readily available because ENE-COVID has stablished a procedure for data request, with a Scientific Board that evaluates these petitions and guarantees the safeguard of participants’ rights, under the limits imposed by the Ethical Committee. Requests should be addressed to ENECOVID@isciii.es.

## Ethics statement

The Institutional Review Committee of the Institute of Health Carlos III approved the study (register number PI 39_2020). All enrolled study participants signed a written informed consent.

## Author contributions

EG-G: analysis and interpretation of data, drafting of the manuscript, statistical analysis, supervision, and critical revision of the manuscript for important intellectual content. MG-S: analysis and interpretation of data, drafting of the manuscript, critical revision of the manuscript for important intellectual content, and supervision. RP-B: acquisition of the data, analysis and interpretation of data, drafting of the manuscript, critical revision of the manuscript for important intellectual content, statistical analysis, and supervision. NF: acquisition of the data, analysis and interpretation of data, drafting of the manuscript, and critical revision of the manuscript for important intellectual content. AR-G: analysis and interpretation of data, critical revision of the manuscript for important intellectual content, and supervision. BA: analysis and interpretation of data, statistical analysis, and critical revision of the manuscript for important intellectual content. IP-R: critical revision of the manuscript for important intellectual content and supervision. MP: acquisition of the data, analysis and interpretation of data, critical revision of the manuscript for important intellectual content, obtaining funding, and supervision. BP-G: acquisition of the data, analysis and interpretation of data, drafting of the manuscript, critical revision of the manuscript for important intellectual content, statistical analysis, obtaining funding, and supervision. All authors contributed to the article and approved the submitted version.

## ENE-COVID study group

Spanish Ministry of Health: Pilar Aparicio Azcárraga; Faustino Blanco; Rodrigo Gutiérrez Fernández; Mariano Martín; Saturnino Mezcua Navarro; Marta Molina; Juan F. Muñoz-Montalvo; Matías Salinero Hernández; Jose L. Sanmartín. Institute of Health Carlos III: Manuel Cuenca-Estrella; José León Paniagua; Raquel Yotti; National Center of Epidemiology: Nerea Fernández de Larrea Baz; Pablo Fernández-Navarro; Roberto Pastor-Barriuso; Beatriz Pérez-Gómez; Marina Pollán; National Center of Microbiology: Ana Avellón; Giovanni Fedele; Aurora Fernández-García; Jesús Oteo Iglesias; María Teresa Pérez Olmeda; National School of Public Health: Israel Cruz; Maria Elena Fernández Martínez; Francisco D. Rodríguez-Cabrera. Harvard T.H. Chan School of Public Health: Miguel A. Hernán. Spanish Regional Health Services: Andalucía: José M. Navarro Marí; Susana Padrones Fernández; Begoña Palop Borrás; Ana Belén Pérez Jiménez; Manuel Rodríguez-Iglesias; José Manuel Rumbao Aguirre. Aragón: Ana María Calvo Gascón; María Luz Lou Alcaine. Asturias: Ignacio Donate Suárez; Mercedes Rodríguez Pérez; Oscar Suárez Álvarez. Baleares: Lluis Carbo Saladrigas; Margarita Cases Sanchís; Adoración Hurtado Fernández; Antonio Oliver; Carlos Javier Villafáfila Gomila. Canarias: José María Barrasa Fernández; Elías Castro Feliciano; María Noemí González Quintana; María Araceli Hernández Betancor; Melisa Hernández Febles; Leopoldo Martín Martín. Cantabria: Inés De Benito Población; Luis-Mariano López López; Teresa Ugarte Miota. Castilla-La Mancha: María Sagrario Celada Pérez; María Natalia Vallés Fernández. Castilla y León: Marta Domínguez-Gil González; Isabel Fernández-Natal; Tomás Maté Enríquez; Gregoria Megías Lobón; Juan Luis Muñoz Bellido; Miguel Villa Arranz. Cataluña: Pilar Ciruela; Maria Doladé Botías; M. Angeles Marcos Maeso; Ariadna Mas i Casals; Dúnia Pérez del Campo. Comunidad Valenciana: Antonio Félix de Castro; Ramón Limón Ramírez. Extremadura: Maria Francisca Elías Retamosa; Manuela Rubio González. Galicia: Antonio Aguilera; María Sinda Blanco Lobeiras; German Bou; Alberto Fuentes Losada. La Rioja: Yolanda Caro; Noemí Marauri; Luis Miguel Soria Blanco. Madrid: Roberto Alonso Fernández; Isabel del Cura González; Montserrat Hernández Pascual; Paloma Merino-Amador. Murcia: Natalia Cabrera Castro; Cristóbal Ramírez Almagro; Manuel Segovia Hernández; Aurora Tomás Lizcano. Navarra: Nieves Ascunce Elizaga; María Ederra Sanz; Carmen Ezpeleta Baquedano. País Vasco: Ana Bustinduy Bascaran; Luis Elorduy Otazua; Susana Iglesias Tamayo. Ceuta: Rebeca Benarroch Benarroch; Jesús Lopera Flores. Melilla: Antonia Vázquez de la Villa.

## Funding

This study was supported by Spanish Ministry of Health, Institute of Health Carlos III, and Spanish National Health System.

## Conflict of interest

The authors declare that the research was conducted in the absence of any commercial or financial relationships that could be construed as a potential conflict of interest.

## Publisher’s note

All claims expressed in this article are solely those of the authors and do not necessarily represent those of their affiliated organizations, or those of the publisher, the editors and the reviewers. Any product that may be evaluated in this article, or claim that may be made by its manufacturer, is not guaranteed or endorsed by the publisher.
