# Supplementary material for: Socio-geographical disparities of obesity and excess weight in adults in Spain: insights from the ENE-COVID study
Source: Front Public Health. 2023 Jul 17;11:1195249. doi: 10.3389/fpubh.2023.1195249 (PMC10387530; doi:10.3389/fpubh.2023.1195249)
Supplement: Supplementary file 1 [file Data_Sheet_1.PDF]

Supplementary Material

Socio-geographical disparities of obesity and excess of weight in adults in Spain: insights from the ENE-COVID study

Enrique Gutiérrez-González, Marta García-Solano, Roberto Pastor-Barriuso, Nerea Fernández de Larrea-Baz, Almudena Rollán-Gordo, Belén Peñalver Argüeso, Isabel Peña-Rey[4], Marina Pollán, Beatriz Pérez-Gómez and the ENE-COVID Study Group

*** Correspondence:**
Beatriz Pérez Gómez bperez@isciii.es

Título del estudio: Encuesta Nacional sero-Epidemiológica de la Infección por SARS-CoV-2 en España **(ENE-COVID).**

Promotores: Ministerio de Sanidad e Instituto de Salud Carlos III

Formulario de Consentimiento Informado PARTICIPANTE ≥ 16 AÑOS. Versión n° 10, 25 de abril de 2020

Este Formulario de Consentimiento Informado consta de dos partes:

- Hoja informativa (para compartir con usted información sobre el estudio).
- Certificado de consentimiento (para firmar si usted está de acuerdo en participar). Dos copias
  - Una para usted
  - La otra para el estudio **ENE-COVID**

Parte 1: Hoja informativa

OBJETIVO DEL ESTUDIO:

Estimado ciudadano/a:

Como ya le han comentado por teléfono a usted o a alguna otra persona de su domicilio, queremos invitarle a participar en el Estudio Nacional Epidemiológico (ENE-COVID), dirigido por el Ministerio de Sanidad y el Instituto de Salud Carlos III (ISCIII), para tener una información más real sobre las dimensiones y la evolución de la epidemia de COVID-19 en España y sobre cuál es el estado inmunológico de la población española en relación con el coronavirus que la causa. Los resultados de este estudio, en el que participarán alrededor de 60.000 personas, serán una información clave para poder ir adaptando las medidas de salud pública relacionadas con el control de esta epidemia. Este estudio está aprobado por el Comité de Ética del ISCIII.

¿POR QUÉ LE INVITAMOS A USTED A PARTICIPAR?

El domicilio en el que usted reside ha sido seleccionado completamente al azar por el Instituto Nacional de Estadística, que nos ha facilitado sus datos identificativos y de contacto. Queremos estudiar a todas las personas que viven en los domicilios seleccionados, para tener participantes de todas las edades y de ambos sexos, y les invitamos a todos a colaborar. Esta forma de selección hace que nuestro estudio pueda dar una imagen más real de la situación de la epidemia en España.

¿EN QUÉ CONSISTE SU PARTICIPACIÓN?

Si usted está de acuerdo en participar, el encuestador le hará algunas preguntas relacionadas con esta enfermedad o con su estado de salud. Además de las preguntas, obtendremos unas gotas de sangre de su dedo mediante un pequeño pinchazo. Con esta sangre haremos un test rápido de detección de anticuerpos frente a este coronavirus (15 minutos aproximadamente).

Además, personal de enfermería del estudio obtendrá una muestra de sangre (venopunción) que se analizará en un laboratorio de su Comunidad Autónoma o en el Centro Nacional de Microbiología (CNM) del ISCIII para medir anticuerpos mediante técnicas de laboratorio que podrían detectar con mayor precisión la existencia de anticuerpos. Si sobrase parte de esta muestra, le pedimos también permiso para almacenarla en el CNM en una colección para estudios relacionados con la COVID-19, cuya responsable es Marina Pollán, investigadora de ENE-COVID, y que será registrada como tal en el Registro Nacional de Biobancos. No se usarán para otro motivo ni por otros investigadores ajenos sin pedirle permiso de nuevo. Le pedimos también que nos permita, más adelante, consultar su historia clínica para incorporar al estudio sus datos clínicos relacionados con COVID-19.

Contactaremos de nuevo con ustedes para repetir el estudio y la toma de muestras dentro de 21 días, de nuevo con una pequeña encuesta. Volveremos a contactar una tercera vez, pasados otros 21 días, para hacer lo mismo. Estas visitas repetidas nos permitirán ir viendo si las personas que antes no tenían anticuerpos frente al virus los han adquirido a lo largo de este tiempo.

SU PARTICIPACIÓN ES TOTALMENTE VOLUNTARIA

Su participación es voluntaria. También puede decidir participar en una visita y luego cambiar de opinión, aunque los datos y resultados obtenidos hasta ese momento seguirán formando parte del estudio por razones de interés público. Simplemente háganos saber que ya no quiere participar, bien a la persona que les contacte para concertar las visitas o a las personas del estudio que tomen las muestras, bien por teléfono en el número que le facilitaremos. Este estudio no tiene ninguna relación con su atención sanitaria.

¿QUÉ OBTENGO DE MI PARTICIPACIÓN?

En este estudio se le va a hacer un test rápido, que detecta si usted tiene anticuerpos, es decir, podrá saber si tiene "defensas" frente al coronavirus que produce la enfermedad COVID-19. En el momento actual no se conoce si tener este tipo de defensas significa estar protegido frente a un nuevo contagio por este coronavirus. Un resultado positivo indica simplemente que ha tenido contacto con el virus.

Lo que estos test no van a hacer es diagnosticar la enfermedad llamada COVID-19 ahora mismo, ni permitirle saber si es o no contagioso/a. Hay personas infectadas, especialmente en los primeros días, que aún no tienen anticuerpos y dan negativo en este test. Tampoco todas las personas con anticuerpos dan positivo, sobre todo si sus niveles de anticuerpos son bajos. Podría haber también casos, probablemente muy pocos, en los que el test fuese positivo por error. De todas maneras, este es un virus nuevo, y aún no entendemos bien cómo se comporta nuestro cuerpo frente a él. Este estudio no está diseñado para realizar diagnósticos clínicos ni proporcionar recomendaciones individualizadas de salud pública a cada participante. La información proporcionada por los test de diagnóstico rápido se utilizará exclusivamente para los objetivos del estudio. Esta información no está destinada a su utilización para establecer diagnósticos de COVID-19, recomendar aislamientos u otras actuaciones que corresponden al ámbito clínico.

Le facilitaremos el resultado de su test, junto con información general de las recomendaciones de salud pública de prevención y control. En este sentido, le aconsejamos que siga siempre las recomendaciones de las autoridades sanitarias. Además, usted recibirá una copia firmada de este formulario.

Gracias a su colaboración, dispondremos de datos epidemiológicos que serán muy útiles para ayudar a controlar esta epidemia.

¿CUÁLES SON LOS RIESGOS DE MI PARTICIPACIÓN?

Quizá note un poco de dolor cuando le pinchemos el dedo para obtener unas gotas de sangre. Es muy poco probable que sufra algún daño físico como consecuencia de esto. La toma de muestras de sangre (venopunción) puede provocar una sensación de ardor en el punto de entrada de la aguja en la piel y le puede ocasionar un pequeño hematoma o una leve infección, que desaparecen en pocos días. Más raramente mareo en el momento de la extracción de sangre. La enfermera o enfermero estará atento a estos efectos. En el caso de que hubiese algún problema, recibiría los mejores cuidados que nuestro sistema público de salud puede proporcionar. No recibirá ninguna compensación económica o material por su participación en el estudio.

¿CÓMO RESPETAREMOS LA CONFIDENCIALIDAD DE SUS DATOS? POLÍTICA DE PRIVACIDAD

No compartiremos su información personal con nadie que no sea parte del equipo de este estudio. Su nombre no aparecerá asociado a ninguna muestra de sangre de las que tomaremos. Su nombre se asociará a un número único que se utilizará para identificar su sangre y la información de la encuesta. No publicaremos ningún resultado de la encuesta con datos individuales, y su nombre no aparecerá en la publicación. Todos los miembros del equipo investigador están comprometidos a proteger la confidencialidad de la información que usted nos proporcione, y están obligados a ello por secreto profesional. Los miembros del Comité de Ética del Instituto de Salud Carlos III y las autoridades sanitarias podrían tener acceso a su información personal, sin embargo todos ellos deben respetar la confidencialidad y su información personal nunca se hará pública.

La recogida y tratamiento de datos personales que se necesitan para este estudio se tratarán de acuerdo al Reglamento (UE) 2016/679, General de Protección de Datos, la Ley Orgánica 3/2018 de protección de datos personales y garantía de los derechos digitales, y a la Ley 14/2007, de 3 de julio de investigación biomédica, así como en los términos previstos sobre el estado de alarma. Los responsables del tratamiento de sus datos y sus destinatarios son el Ministerio de Sanidad y el Instituto de Salud Carlos III, ante quienes puede usted ejercer sus derechos de acceso, rectificación, supresión, limitación, oposición a su tratamiento y portabilidad de los datos. La información se usará con su consentimiento y/o por interés público en el ámbito de la salud pública, o el cumplimiento de obligaciones legales. Sus datos se conservarán el tiempo necesario para las finalidades que se han descrito.

Si considera vulnerados sus derechos puede dirigirse a los delegados de protección de datos del Ministerio de Sanidad y del ISCIII mediante un correo electrónico (delegadoprotecciondatos@mscbs.es; dpd@isciii.es), o mediante correo postal, o interponer una reclamación ante la Agencia Española de Protección de Datos (AEPD) (C/Jorge Juan, 6. 28001. Madrid. www.aepd.es).

¿Con quién contactar si tiene algún problema o pregunta?

Por favor, antes de firmar el documento de consentimiento, haga todas las preguntas que necesite a los entrevistadores. Ellos tienen que aclararle todas sus dudas. Para cualquier problema o pregunta en relación a este estudio usted puede también contactar a: Raquel Yotti o Marina Pollán (Investigadoras principales de ENE-COVID) I Email de contacto: ene-covid@isciii.es. ISCIII. Monforte de Lemos 5 – 28029 Madrid.

Parte 2: Certificado de Consentimiento: COPIA PARA EL PARTICIPANTE

Para PARTICIPANTE ≥ 16 AÑOS que pueda leer el formulario de consentimiento informado

Necesitamos que nos dé su consentimiento para participar en el estudio y para usar sus datos personales. Además, le vamos a pedir otros permisos que no son imprescindibles para ello. Puede, libremente, escoger decir que sí o no, sin que ello afecte su participación en este estudio.

Consentimiento informado

- He leído la hoja informativa de la encuesta, o un encuestador o un miembro de mi familia me la ha leído. He comprendido para qué es la encuesta y en qué consiste mi participación, así como los riesgos y beneficios de la misma. He podido preguntar cualquier duda y me las han respondido de manera satisfactoria. Acepto participar en esta encuesta de manera voluntaria. Entiendo también que mi sangre podría ser almacenada por algún tiempo y enviada a otros laboratorios diferentes a los del Instituto de Salud Carlos III para realizar otros estudios en relación con COVID-19.

Acepto participar en **la encuesta sero-epidemiológica,** contestando la encuesta epidemiológica y la realización del test rápido**.** Además, he comprendido la **información sobre el uso de mis datos personales** y consiento — ☐ **SÍ** ☐ **NO**

- Acepto dar una muestra de sangre para medir anticuerpos con las técnicas que se están desarrollando — ☐ SÍ ☐ NO
- Estoy de acuerdo con que parte de esa sangre, si sobra, se almacene en una colección de muestras para estudios relacionados con la COVID-19. — ☐ SÍ ☐ NO

  (*Si no consiento, será destruida una vez finalizado el estudio*).

- Autorizo que me contacten de nuevo para pedirme permiso si otros investigadores u otras líneas de investigación quisieran usar las muestras — ☐ SÍ ☐ NO
- Doy permiso para que personal del estudio pueda, más adelante, consultar la información de mi historia clínica para incorporar al estudio los datos clínicos relacionados con COVID-19, y entiendo que esto no forma parte de mi asistencia médica — ☐ SÍ ☐ NO

NOMBRE y APELLIDOS del / de la participante:

FECHA

FIRMA

Para testigos de PARTICIPANTE ≥ 16 AÑOS que no puede leer el formulario de consentimiento informado:

He sido testigo de la lectura precisa del formulario de consentimiento, y el/la participante ha tenido la oportunidad de preguntar dudas y estas le han sido aclaradas. Confirmo que el/la participante da su consentimiento libremente.

NOMBRE y APELLIDOS del /de la testigo____

Y huella digital del/ de la participante:

FIRMA del/ de la testigo ____________________________________

Fecha ________________________ día/mes/año

Parte 2: Certificado de Consentimiento PARA EL ESTUDIO: PONER PEGATINA CODIGO ESTUDIO

Para PARTICIPANTE ≥ 16 AÑOS que pueda leer el formulario de consentimiento informado

Necesitamos que nos dé su consentimiento para participar en el estudio y para usar sus datos personales. Además, le vamos a pedir otros permisos que no son imprescindibles para ello. Puede, libremente escoger decir que sí o no, sin que ello afecte su participación en este estudio.

Consentimiento informado

- He leído la hoja informativa de la encuesta, o un encuestador o un miembro de mi familia me la ha leído. He comprendido para qué es la encuesta y en qué consiste mi participación, así como los riesgos y beneficios de la misma. He podido preguntar cualquier duda y me las han respondido de manera satisfactoria. Acepto participar en esta encuesta de manera voluntaria. Entiendo también que mi sangre podría ser almacenada por algún tiempo y enviada a otros laboratorios diferentes a los del Instituto de Salud Carlos III para realizar otros estudios en relación con COVID-19.

Acepto participar en **la encuesta sero-epidemiológica,** contestando la encuesta epidemiológica y la realización del test rápido**.** Además, he comprendido la **información sobre el uso de mis datos personales** y consiento — ☐ **SÍ** ☐ **NO**

- Acepto dar una muestra de sangre para medir anticuerpos con las técnicas que se están desarrollando — ☐ SÍ ☐ NO
- Estoy de acuerdo con que parte de esa sangre, si sobra, se almacene en una colección de muestras para estudios relacionados con la COVID-19. — ☐ SÍ ☐ NO

  (*Si no consiento, será destruida una vez finalizado el estudio*).

- Autorizo que me contacten de nuevo para pedirme permiso si otros investigadores u otras líneas de investigación quisieran usar las muestras — ☐ SÍ ☐ NO
- Doy permiso para que personal del estudio pueda, más adelante, consultar la información de mi historia clínica para incorporar al estudio los datos clínicos relacionados con COVID-19, y entiendo que esto no forma parte de mi asistencia médica — ☐ SÍ ☐ NO

NOMBRE y APELLIDOS del / de la participante:

FECHA

FIRMA

Para testigos de PARTICIPANTE ≥ 16 AÑOS que no puede leer el formulario de consentimiento informado:

He sido testigo de la lectura precisa del formulario de consentimiento, y el/la participante ha tenido la oportunidad de preguntar dudas y estas le han sido aclaradas. Confirmo que el/la participante da su consentimiento libremente.

NOMBRE y APELLIDOS del /de la testigo____

Y huella digital del/ de la participante:

FIRMA del/ de la testigo _______________________________________

Fecha _________________________ día/mes/año
