# Supplementary material for: Socio-geographical disparities of obesity and excess weight in adults in Spain: insights from the ENE-COVID study
Source: Front Public Health. 2023 Jul 17;11:1195249. doi: 10.3389/fpubh.2023.1195249 (PMC10387530; doi:10.3389/fpubh.2023.1195249)

Supplementary Material

Socio-geographical disparities of obesity and excess of weight in adults in Spain: insights from the ENE-COVID study

Enrique Gutiérrez-González, Marta García-Solano, Roberto Pastor-Barriuso, Nerea Fernández de Larrea-Baz, Almudena Rollán-Gordo, Belén Peñalver Argüeso, Isabel Peña-Rey[4], Marina Pollán, Beatriz Pérez-Gómez and the ENE-COVID Study Group

*** Correspondence:**
Beatriz Pérez Gómez bperez@isciii.es

Supplementary Figure S3. Age-standardized prevalences (%) of obesity and excess weight by province and sex in adult participants from ENE-COVID study

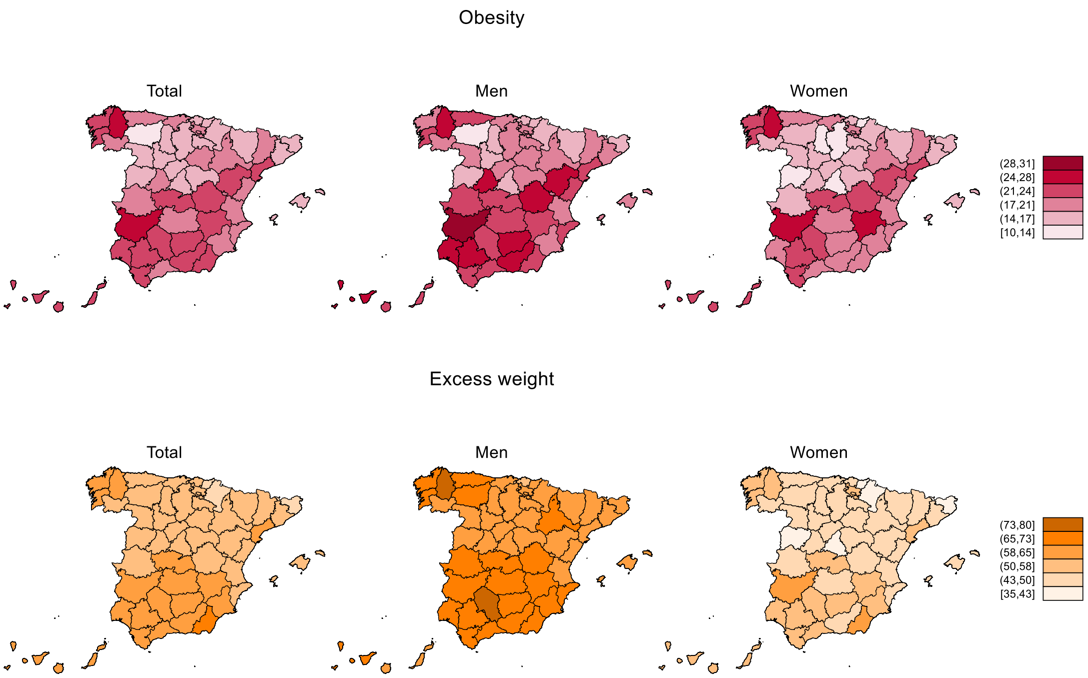

Supplement: Supplementary file 4 [file Image_3.PDF]
