# Supplementary material for: Socio-geographical disparities of obesity and excess weight in adults in Spain: insights from the ENE-COVID study
Source: Front Public Health. 2023 Jul 17;11:1195249. doi: 10.3389/fpubh.2023.1195249 (PMC10387530; doi:10.3389/fpubh.2023.1195249)
Supplement: Supplementary file 5 [file Table_1.DOCX]

Supplementary Material

Socio-geographical disparities of obesity and excess of weight in adults in Spain: insights from the ENE-COVID study

**Enrique Gutiérrez-González, Marta García-Solano, Roberto Pastor-Barriuso, Nerea Fernández de Larrea-Baz, Almudena Rollán-Gordo, Belén Peñalver Argüeso, Isabel Peña-Rey^4^, Marina Pollán, Beatriz Pérez-Gómez and the ENE-COVID Study Group**

*** Correspondence:**Beatriz Pérez Gómez [bperez@isciii.es](mailto:bperez@isciii.es)

**Supplementary Table S1**. crude prevalence of severe obesity in adult population by sociodemographic characteristics in ENE-COVID study

|  | **TOTAL** | | **MEN** | | **WOMEN** | |
| --- | --- | --- | --- | --- | --- | --- |
|  | **N** | **% (95% CI)** | **N** | **% (95% CI)** | **N** | **% (95% CI)** |
| **SEVERE OBESITY (BMI≥35 kg/m^2^)** |  |  |  |  |  |  |
| **Overall** | 57131 | 4.9 (4.6-5.1) | 27031 | 4.5 (4.1-4.8) | 30100 | 5.3 (5.0-5.6) |
| **Age (years)** |  |  |  |  |  |  |
| 18-24 | 4601 | 2.0 (1.6-2.6) | 2267 | 2.3 (1.6-3.3) | 2334 | 1.7 (1.2-2.5) |
| 25-29 | 2991 | 2.8 (2.2-3.6) | 1451 | 1.6 (1.0-2.4) | 1540 | 4.1 (3.1-5.5) |
| 30-34 | 3404 | 4.1 (3.3-5.0) | 1617 | 4.3 (3.2-5.9) | 1787 | 3.8 (2.9-5.1) |
| 35-39 | 4380 | 4.1 (3.4-4.9) | 2112 | 3.8 (2.9-5.0) | 2268 | 4.4 (3.5-5.5) |
| 40-44 | 5646 | 4.9 (4.3-5.7) | 2707 | 5.0 (4.0-6.2) | 2939 | 4.9 (4.0-5.9) |
| 45-49 | 6018 | 4.7 (4.1-5.4) | 2902 | 4.4 (3.6-5.4) | 3116 | 5.0 (4.0-6.2) |
| 50-54 | 5846 | 5.8 (5.1-6.6) | 2748 | 6.0 (5.0-7.2) | 3098 | 5.6 (4.7-6.6) |
| 55-59 | 5749 | 6.1 (5.4-7.0) | 2676 | 5.7 (4.7-6.8) | 3073 | 6.6 (5.6-7.7) |
| 60-64 | 5200 | 6.7 (5.8-7.7) | 2512 | 5.9 (4.9-7.3) | 2688 | 7.5 (6.2-9.0) |
| 65-69 | 4029 | 6.3 (5.4-7.3) | 1961 | 5.3 (4.2-6.7) | 2068 | 7.2 (5.9-8.7) |
| 70-74 | 3386 | 6.1 (5.2-7.1) | 1582 | 5.8 (4.5-7.4) | 1804 | 6.3 (5.2-7.7) |
| 75-79 | 2477 | 6.3 (5.2-7.6) | 1142 | 4.7 (3.3-6.7) | 1335 | 7.5 (5.9-9.4) |
| 80-84 | 1701 | 5.2 (4.0-6.8) | 710 | 3.0 (1.8-5.1) | 991 | 6.6 (4.9-8.9) |
| 85-89 | 1140 | 3.2 (2.1-4.8) | 449 | 1.4 (0.6-3.6) | 691 | 4.2 (2.6-6.7) |
| ≥90 | 563 | 3.6 (2.3-5.8) | 195 | 2.5 (0.8-7.5) | 368 | 4.2 (2.5-6.9) |
| **Education** |  |  |  |  |  |  |
| Less than primary | 3678 | 9.6 (8.5-10.8) | 1490 | 7.8 (6.3-9.6) | 2188 | 10.7 (9.3-12.3) |
| Primary | 7811 | 7.0 (6.3-7.8) | 3523 | 5.4 (4.6-6.3) | 4288 | 8.4 (7.4-9.5) |
| Secondary | 31164 | 4.9 (4.6-5.2) | 15674 | 4.7 (4.2-5.1) | 15490 | 5.1 (4.7-5.6) |
| University | 12587 | 2.6 (2.3-2.9) | 5352 | 2.7 (2.2-3.3) | 7235 | 2.5 (2.1-2.9) |
| **Census tract average income*** |  |  |  |  |  |  |
| <25^th^ percentile | 15229 | 6.2 (5.7-6.8) | 7230 | 5.3 (4.6-6.0) | 7999 | 7.1 (6.4-7.9) |
| 25^th^-<50^th^ percentile | 14397 | 5.1 (4.7-5.6) | 6838 | 4.9 (4.3-5.6) | 7559 | 5.3 (4.7-5.9) |
| 50^th^-<75^th^ percentile | 13054 | 4.7 (4.2-5.3) | 6221 | 4.1 (3.5-4.8) | 6833 | 5.3 (4.7-6.1) |
| ≥75^th^ percentile | 14451 | 3.4 (3.0-3.9) | 6742 | 3.5 (2.9-4.2) | 7709 | 3.4 (2.9-4.0) |
| **Disability** |  |  |  |  |  |  |
| No | 52224 | 4.7 (4.4-4.9) | 24468 | 4.3 (4.0-4.7) | 27756 | 5.0 (4.7-5.3) |
| Yes | 3171 | 8.5 (7.5-9.7) | 1660 | 6.4 (5.1-8.0) | 1511 | 10.9 (9.2-12.9) |
| **Nationality** |  |  |  |  |  |  |
| Spanish | 54559 | 4.9 (4.7-5.2) | 25947 | 4.5 (4.2-4.9) | 28612 | 5.3 (5.0-5.6) |
| Other | 2562 | 4.3 (3.4-5.4) | 1080 | 3.3 (2.3-4.9) | 1482 | 5.0 (3.8-6.7) |
| **Municipality size (inhabitants)** |  |  |  |  |  |  |
| <5,000 | 10401 | 5.8 (5.2-6.5) | 5160 | 5.4 (4.6-6.3) | 5241 | 6.3 (5.4-7.3) |
| 5,000-19,999 | 12045 | 5.3 (4.8-5.8) | 5730 | 4.8 (4.2-5.5) | 6315 | 5.8 (5.1-6.6) |
| 20,000-99,999 | 17006 | 4.7 (4.3-5.2) | 7939 | 4.2 (3.7-4.9) | 9067 | 5.1 (4.6-5.7) |
| ≥100,000 | 17679 | 4.6 (4.2-5.0) | 8202 | 4.2 (3.6-4.8) | 9477 | 4.9 (4.4-5.5) |

Population prevalence and 95% confidence intervals (CI) accounting for sampling weights, nonresponse rates by sex, age, and census tract average income, stratification by province and municipality size, and clustering by household and census tract. BMI: Body Mass Index; N: number of participants; *Categories based on percentiles from province-specific distributions of census tract average income in 2017.
