# Supplementary material for: Socio-geographical disparities of obesity and excess weight in adults in Spain: insights from the ENE-COVID study
Source: Front Public Health. 2023 Jul 17;11:1195249. doi: 10.3389/fpubh.2023.1195249 (PMC10387530; doi:10.3389/fpubh.2023.1195249)
Supplement: Supplementary file 6 [file Table_2.DOCX]

Supplementary Material

Socio-geographical disparities of obesity and excess of weight in adults in Spain: insights from the ENE-COVID study

**Enrique Gutiérrez-González, Marta García-Solano, Roberto Pastor-Barriuso, Nerea Fernández de Larrea-Baz, Almudena Rollán-Gordo, Belén Peñalver Argüeso, Isabel Peña-Rey^4^, Marina Pollán, Beatriz Pérez-Gómez and the ENE-COVID Study Group**

*** Correspondence:**Beatriz Pérez Gómez [bperez@isciii.es](mailto:bperez@isciii.es)

**Supplementary Table S2**. Crude prevalence of obesity and excess weight by age group and sex in adult population in ENE-COVID study

|  | **TOTAL** | | **MEN** | | **WOMEN** | |
| --- | --- | --- | --- | --- | --- | --- |
|  | **N** | **% (95% CI)** | **N** | **% (95% CI)** | **N** | **% (95% CI)** |
| **OBESITY** |  |  |  |  |  |  |
| **Age (years)** |  |  |  |  |  |  |
| 18-24 | 4601 | 6.9 (6.0-7.9) | 2267 | 7.5 (6.2-9.0) | 2334 | 6.3 (5.1-7.6) |
| 25-29 | 2991 | 10.2 (8.9-11.6) | 1451 | 10.0 (8.1-12.1) | 1540 | 10.4 (8.7-12.4) |
| 30-34 | 3404 | 13.7 (12.2-15.3) | 1617 | 14.4 (12.3-16.8) | 1787 | 12.9 (11.1-15.1) |
| 35-39 | 4380 | 14.4 (13.1-15.8) | 2112 | 14.7 (12.9-16.7) | 2268 | 14.1 (12.4-16.0) |
| 40-44 | 5646 | 16.6 (15.4-17.9) | 2707 | 18.8 (16.9-20.8) | 2939 | 14.5 (13.0-16.1) |
| 45-49 | 6018 | 18.8 (17.6-20.1) | 2902 | 20.4 (18.6-22.2) | 3116 | 17.1 (15.5-18.9) |
| 50-54 | 5846 | 21.0 (19.7-22.5) | 2748 | 23.9 (22.0-26.0) | 3098 | 18.2 (16.5-20.0) |
| 55-59 | 5749 | 22.8 (21.4-24.2) | 2676 | 24.9 (22.9-26.9) | 3073 | 20.9 (19.1-22.8) |
| 60-64 | 5200 | 24.3 (22.7-25.9) | 2512 | 26.3 (24.2-28.5) | 2688 | 22.2 (20.2-24.4) |
| 65-69 | 4029 | 25.8 (24.1-27.5) | 1961 | 26.8 (24.4-29.4) | 2068 | 24.9 (22.5-27.3) |
| 70-74 | 3386 | 26.5 (24.7-28.4) | 1582 | 27.8 (25.0-30.7) | 1804 | 25.5 (23.3-27.9) |
| 75-79 | 2477 | 25.0 (22.8-27.4) | 1142 | 21.1 (18.2-24.4) | 1335 | 28.0 (25.1-31.2) |
| 80-84 | 1701 | 25.6 (23.0-28.4) | 710 | 21.3 (17.9-25.1) | 991 | 28.3 (25.0-31.9) |
| 85-89 | 1140 | 22.2 (19.2-25.6) | 449 | 18.3 (14.1-23.4) | 691 | 24.6 (20.7-28.9) |
| ≥90 | 563 | 17.7 (14.1-22.0) | 195 | 11.5 (7.1-18.2) | 368 | 20.7 (16.0-26.3) |
| **EXCESS WEIGHT** |  |  |  |  |  |  |
| **Age (years)** |  |  |  |  |  |  |
| 18-24 | 4601 | 24.1 (22.4-25.8) | 2267 | 27.6 (25.3-30.1) | 2334 | 20.4 (18.4-22.6) |
| 25-29 | 2991 | 37.0 (34.8-39.4) | 1451 | 43.1 (39.8-46.5) | 1540 | 30.9 (28.0-34.0) |
| 30-34 | 3404 | 44.2 (42.0-46.5) | 1617 | 52.8 (49.6-55.9) | 1787 | 35.6 (32.7-38.6) |
| 35-39 | 4380 | 50.3 (48.3-52.2) | 2112 | 60.3 (57.6-63.0) | 2268 | 40.2 (37.6-42.9) |
| 40-44 | 5646 | 52.9 (51.2-54.5) | 2707 | 65.0 (62.9-67.1) | 2939 | 40.6 (38.3-42.9) |
| 45-49 | 6018 | 56.9 (55.3-58.5) | 2902 | 67.7 (65.5-69.9) | 3116 | 45.6 (43.3-47.9) |
| 50-54 | 5846 | 61.1 (59.4-62.7) | 2748 | 71.9 (69.7-74.0) | 3098 | 50.5 (48.2-52.8) |
| 55-59 | 5749 | 64.6 (62.9-66.1) | 2676 | 75.9 (73.8-77.9) | 3073 | 54.3 (52.0-56.6) |
| 60-64 | 5200 | 68.3 (66.7-69.8) | 2512 | 77.2 (75.1-79.2) | 2688 | 59.3 (56.9-61.6) |
| 65-69 | 4029 | 71.4 (69.5-73.2) | 1961 | 76.9 (74.6-79.1) | 2068 | 66.6 (63.9-69.2) |
| 70-74 | 3386 | 70.6 (68.5-72.6) | 1582 | 78.0 (75.5-80.3) | 1804 | 64.8 (61.7-67.8) |
| 75-79 | 2477 | 72.1 (69.6-74.5) | 1142 | 74.7 (71.3-77.8) | 1335 | 70.1 (66.7-73.2) |
| 80-84 | 1701 | 71.9 (68.8-74.8) | 710 | 76.1 (71.9-79.9) | 991 | 69.2 (65.3-72.9) |
| 85-89 | 1140 | 65.3 (61.5-68.9) | 449 | 69.7 (63.7-75.1) | 691 | 62.7 (58.0-67.2) |
| ≥90 | 563 | 58.7 (53.4-63.9) | 195 | 62.7 (53.2-71.3) | 368 | 56.8 (50.0-63.4) |
