# Supplementary material for: Socio-geographical disparities of obesity and excess weight in adults in Spain: insights from the ENE-COVID study
Source: Front Public Health. 2023 Jul 17;11:1195249. doi: 10.3389/fpubh.2023.1195249 (PMC10387530; doi:10.3389/fpubh.2023.1195249)
Supplement: Supplementary file 7 [file Table_3.DOCX]

Supplementary Material

Socio-geographical disparities of obesity and excess of weight in adults in Spain: insights from the ENE-COVID study

**Enrique Gutiérrez-González, Marta García-Solano, Roberto Pastor-Barriuso, Nerea Fernández de Larrea-Baz, Almudena Rollán-Gordo, Belén Peñalver Argüeso, Isabel Peña-Rey^4^, Marina Pollán, Beatriz Pérez-Gómez and the ENE-COVID Study Group**

*** Correspondence:**Beatriz Pérez Gómez [bperez@isciii.es](mailto:bperez@isciii.es)

**Supplementary Table S3**. Crude prevalence of obesity by sex and province in adults in ENE-COVID study

|  | **TOTAL** | | **MEN** | | **WOMEN** | |
| --- | --- | --- | --- | --- | --- | --- |
|  | **N** | **% (95% CI)** | **N** | **% (95% CI)** | **N** | **% (95% CI)** |
| Spain | 57131 | 18.7 (18.1-19.2) | 27031 | 19.3 (18.7-20.0) | 30100 | 18.0 (17.4-18.7) |
| **Province** |  |  |  |  |  |  |
| Albacete | 943 | 23.2 (18.9-28.0) | 459 | 21.3 (17.5-25.6) | 484 | 25.0 (18.4-33.1) |
| Alicante/Alacant | 1507 | 19.5 (17.0-22.3) | 714 | 21.2 (17.6-25.3) | 793 | 17.9 (15.0-21.1) |
| Almería | 844 | 19.9 (15.7-24.8) | 393 | 21.2 (15.5-28.3) | 451 | 18.5 (14.9-22.7) |
| Araba/Álava | 693 | 18.2 (15.1-21.7) | 333 | 18.1 (14.3-22.7) | 360 | 18.2 (13.6-24.0) |
| Asturias | 1604 | 21.2 (19.1-23.5) | 733 | 22.3 (19.4-25.4) | 871 | 20.3 (17.5-23.4) |
| Ávila | 619 | 20.5 (17.2-24.4) | 306 | 26.4 (21.3-32.2) | 313 | 14.7 (11.6-18.6) |
| Badajoz | 1415 | 27.0 (23.0-31.5) | 705 | 27.4 (22.8-32.6) | 710 | 26.7 (22.4-31.4) |
| Balears, Illes | 1214 | 16.0 (13.9-18.3) | 579 | 16.9 (14.3-19.9) | 635 | 15.0 (12.6-17.8) |
| Barcelona | 3307 | 16.6 (14.8-18.5) | 1536 | 16.9 (14.8-19.3) | 1771 | 16.2 (14.1-18.5) |
| Bizkaia | 1181 | 16.9 (13.2-21.5) | 550 | 16.9 (13.9-20.5) | 631 | 16.9 (11.9-23.5) |
| Burgos | 793 | 15.0 (12.4-18.0) | 391 | 18.6 (14.7-23.2) | 402 | 11.4 (8.7-14.9) |
| Cáceres | 1083 | 19.1 (16.1-22.5) | 528 | 20.9 (17.7-24.4) | 555 | 17.4 (13.4-22.2) |
| Cádiz | 1235 | 21.4 (18.7-24.4) | 572 | 20.2 (16.8-24.2) | 663 | 22.6 (18.7-27.1) |
| Cantabria | 1480 | 18.6 (15.4-22.2) | 713 | 18.9 (15.7-22.5) | 767 | 18.3 (14.1-23.5) |
| Castellón/Castelló | 754 | 20.1 (16.7-24.0) | 361 | 20.9 (16.6-26.1) | 393 | 19.3 (14.9-24.6) |
| Ciudad Real | 1034 | 20.4 (16.9-24.3) | 493 | 23.0 (18.4-28.5) | 541 | 17.8 (14.2-22.0) |
| Córdoba | 963 | 23.2 (19.4-27.6) | 444 | 23.1 (19.1-27.5) | 519 | 23.4 (18.5-29.2) |
| Coruña, A | 1193 | 22.5 (19.4-25.9) | 548 | 21.1 (15.8-27.6) | 645 | 23.7 (19.9-27.9) |
| Cuenca | 753 | 24.4 (20.6-28.7) | 382 | 25.7 (21.4-30.5) | 371 | 23.1 (18.5-28.6) |
| Gipuzkoa | 934 | 14.7 (11.1-19.1) | 450 | 15.3 (11.0-20.8) | 484 | 14.1 (10.7-18.3) |
| Girona | 1022 | 13.9 (11.2-17.1) | 457 | 13.3 (10.1-17.3) | 565 | 14.5 (11.2-18.6) |
| Granada | 945 | 22.1 (19.3-25.1) | 437 | 26.5 (21.6-32.0) | 508 | 18.0 (13.3-23.9) |
| Guadalajara | 742 | 16.5 (13.6-19.8) | 359 | 18.0 (13.5-23.5) | 383 | 14.9 (11.7-18.9) |
| Huelva | 855 | 21.2 (17.2-25.8) | 393 | 23.2 (17.0-30.9) | 462 | 19.2 (16.1-22.7) |
| Huesca | 659 | 15.8 (12.8-19.3) | 301 | 14.1 (10.4-18.8) | 358 | 17.5 (13.1-23.1) |
| Jaén | 921 | 22.0 (19.5-24.7) | 433 | 25.0 (21.2-29.3) | 488 | 19.1 (15.6-23.1) |
| León | 868 | 14.1 (11.7-17.0) | 408 | 14.0 (10.4-18.5) | 460 | 14.3 (11.0-18.3) |
| Lleida | 709 | 19.1 (15.4-23.3) | 336 | 18.7 (14.8-23.3) | 373 | 19.5 (13.9-26.5) |
| Lugo | 743 | 27.3 (22.1-33.3) | 353 | 26.5 (21.4-32.3) | 390 | 28.1 (21.2-36.3) |
| Madrid | 3358 | 14.6 (13.1-16.2) | 1595 | 16.2 (14.3-18.3) | 1763 | 13.1 (11.3-15.2) |
| Málaga | 1246 | 20.1 (16.4-24.4) | 592 | 20.4 (15.6-26.2) | 654 | 19.9 (15.7-24.8) |
| Murcia | 1392 | 19.1 (16.4-22.1) | 643 | 19.4 (15.6-23.7) | 749 | 18.8 (15.6-22.4) |
| Navarra | 1519 | 15.8 (13.1-18.8) | 750 | 16.2 (13.5-19.4) | 769 | 15.3 (12.0-19.2) |
| Ourense | 749 | 18.6 (15.5-22.1) | 335 | 21.8 (17.9-26.1) | 414 | 15.7 (12.1-20.2) |
| Palencia | 704 | 15.1 (11.2-20.1) | 335 | 17.8 (12.8-24.2) | 369 | 12.6 (9.0-17.4) |
| Palmas, Las | 1427 | 22.2 (19.1-25.6) | 655 | 21.2 (17.3-25.7) | 772 | 23.1 (20.1-26.4) |
| Pontevedra | 1177 | 22.2 (19.1-25.6) | 551 | 21.1 (17.6-25.2) | 626 | 23.1 (19.0-27.9) |
| Rioja, La | 1213 | 17.0 (14.5-19.8) | 592 | 17.5 (13.9-21.8) | 621 | 16.5 (13.3-20.3) |
| Salamanca | 751 | 14.7 (11.5-18.5) | 342 | 16.4 (12.3-21.6) | 409 | 13.1 (9.4-18.1) |
| Santa Cruz de Tenerife | 1229 | 23.0 (20.7-25.3) | 568 | 23.8 (20.1-27.9) | 661 | 22.1 (18.7-26.0) |
| Segovia | 643 | 17.2 (13.2-22.1) | 316 | 17.8 (13.5-23.1) | 327 | 16.6 (11.1-23.9) |
| Sevilla | 1531 | 23.1 (20.5-26.0) | 726 | 24.8 (21.2-28.9) | 805 | 21.6 (18.5-25.0) |
| Soria | 627 | 18.5 (14.9-22.8) | 315 | 20.0 (15.7-25.1) | 312 | 17.0 (12.9-22.1) |
| Tarragona | 870 | 22.0 (18.3-26.2) | 419 | 21.4 (17.7-25.5) | 451 | 22.7 (18.0-28.2) |
| Teruel | 594 | 23.1 (20.4-25.9) | 302 | 24.4 (18.8-31.0) | 292 | 21.7 (18.0-25.9) |
| Toledo | 1040 | 22.0 (18.4-26.0) | 503 | 21.6 (17.3-26.6) | 537 | 22.3 (17.9-27.6) |
| Valencia/València | 1834 | 17.6 (15.9-19.3) | 865 | 17.1 (14.4-20.2) | 969 | 18.0 (15.6-20.7) |
| Valladolid | 931 | 15.2 (12.1-18.9) | 435 | 15.0 (11.1-19.9) | 496 | 15.4 (11.1-21.0) |
| Zamora | 608 | 18.4 (14.0-23.8) | 300 | 18.8 (14.0-24.6) | 308 | 18.0 (12.0-26.0) |
| Zaragoza | 1170 | 18.7 (16.1-21.7) | 549 | 19.3 (16.4-22.6) | 621 | 18.2 (14.5-22.6) |
| Ceuta | 774 | 18.2 (15.1-21.7) | 346 | 15.4 (12.4-18.9) | 428 | 21.1 (16.4-26.6) |
| Melilla | 731 | 19.5 (16.9-22.3) | 330 | 18.2 (14.9-22.0) | 401 | 20.9 (17.1-25.2) |
