# Supplementary material for: Socio-geographical disparities of obesity and excess weight in adults in Spain: insights from the ENE-COVID study
Source: Front Public Health. 2023 Jul 17;11:1195249. doi: 10.3389/fpubh.2023.1195249 (PMC10387530; doi:10.3389/fpubh.2023.1195249)
Supplement: Supplementary file 8 [file Table_4.DOCX]

Supplementary Material

Socio-geographical disparities of obesity and excess of weight in adults in Spain: insights from the ENE-COVID study

**Enrique Gutiérrez-González, Marta García-Solano, Roberto Pastor-Barriuso, Nerea Fernández de Larrea-Baz, Almudena Rollán-Gordo, Belén Peñalver Argüeso, Isabel Peña-Rey^4^, Marina Pollán, Beatriz Pérez-Gómez and the ENE-COVID Study Group**

*** Correspondence:**Beatriz Pérez Gómez [bperez@isciii.es](mailto:bperez@isciii.es)

**Supplementary Table S4**. Crude prevalence of excess weight by sex and province in adults in ENE-COVID study

|  | **TOTAL** | | **MEN** | | **WOMEN** | |
| --- | --- | --- | --- | --- | --- | --- |
|  | **N** | **% (95% CI)** | **N** | **% (95% CI)** | **N** | **% (95% CI)** |
| Spain | 57131 | 55.8 (55.1-56.4) | 27031 | 63.7 (62.9-64.4) | 30100 | 48.4 (47.5-49.3) |
| **Province** |  |  |  |  |  |  |
| Albacete | 943 | 61.5 (54.6-67.9) | 459 | 69.7 (64.6-74.3) | 484 | 53.4 (44.1-62.4) |
| Alicante/Alacant | 1507 | 55.5 (52.5-58.4) | 714 | 64.3 (60.0-68.4) | 793 | 46.8 (43.3-50.3) |
| Almería | 844 | 64.0 (57.6-69.9) | 393 | 69.7 (62.6-75.9) | 451 | 58.2 (51.3-64.8) |
| Araba/Álava | 693 | 56.4 (50.4-62.1) | 333 | 62.1 (54.4-69.1) | 360 | 51.1 (45.0-57.2) |
| Asturias | 1604 | 59.7 (56.8-62.5) | 733 | 68.1 (65.0-71.0) | 871 | 52.3 (48.5-56.1) |
| Ávila | 619 | 57.1 (52.8-61.4) | 306 | 66.7 (60.7-72.2) | 313 | 47.7 (42.4-53.1) |
| Badajoz | 1415 | 64.8 (62.2-67.4) | 705 | 70.3 (67.2-73.3) | 710 | 59.5 (55.4-63.6) |
| Balears, Illes | 1214 | 52.2 (48.9-55.4) | 579 | 61.4 (56.2-66.3) | 635 | 43.2 (39.3-47.2) |
| Barcelona | 3307 | 52.7 (50.0-55.3) | 1536 | 60.2 (57.7-62.7) | 1771 | 45.8 (42.2-49.4) |
| Bizkaia | 1181 | 53.0 (48.5-57.4) | 550 | 58.3 (53.5-62.8) | 631 | 48.2 (42.1-54.4) |
| Burgos | 793 | 55.6 (49.6-61.5) | 391 | 62.9 (56.9-68.5) | 402 | 48.4 (40.8-56.0) |
| Cáceres | 1083 | 57.7 (53.4-61.9) | 528 | 66.7 (62.2-70.8) | 555 | 48.9 (43.1-54.8) |
| Cádiz | 1235 | 60.6 (58.1-63.0) | 572 | 66.9 (62.6-70.9) | 663 | 54.4 (49.6-59.2) |
| Cantabria | 1480 | 55.7 (52.1-59.4) | 713 | 62.5 (57.6-67.2) | 767 | 49.4 (44.7-54.2) |
| Castellón/Castelló | 754 | 55.7 (51.4-59.9) | 361 | 63.8 (58.4-68.9) | 393 | 48.0 (40.9-55.2) |
| Ciudad Real | 1034 | 57.7 (53.6-61.8) | 493 | 65.3 (59.7-70.5) | 541 | 50.5 (46.1-55.0) |
| Córdoba | 963 | 63.4 (58.6-68.0) | 444 | 72.7 (68.6-76.5) | 519 | 54.7 (47.3-61.9) |
| Coruña, A | 1193 | 62.6 (58.4-66.6) | 548 | 67.5 (62.2-72.3) | 645 | 58.1 (53.3-62.8) |
| Cuenca | 753 | 58.0 (53.3-62.5) | 382 | 66.3 (60.5-71.7) | 371 | 49.6 (43.9-55.3) |
| Gipuzkoa | 934 | 51.2 (47.2-55.2) | 450 | 59.0 (54.3-63.5) | 484 | 43.9 (38.1-49.8) |
| Girona | 1022 | 47.9 (44.6-51.2) | 457 | 57.2 (53.1-61.2) | 565 | 38.9 (34.0-44.1) |
| Granada | 945 | 57.8 (53.1-62.3) | 437 | 67.0 (61.7-71.8) | 508 | 49.2 (41.7-56.6) |
| Guadalajara | 742 | 52.7 (48.8-56.6) | 359 | 61.2 (55.5-66.7) | 383 | 44.0 (38.2-49.9) |
| Huelva | 855 | 62.3 (57.4-67.0) | 393 | 70.1 (64.3-75.4) | 462 | 54.7 (49.1-60.3) |
| Huesca | 659 | 55.2 (50.1-60.1) | 301 | 65.4 (60.1-70.3) | 358 | 45.0 (38.3-51.9) |
| Jaén | 921 | 61.0 (56.8-65.1) | 433 | 65.5 (60.4-70.3) | 488 | 56.7 (51.5-61.7) |
| León | 868 | 57.9 (52.9-62.8) | 408 | 68.1 (61.6-74.0) | 460 | 49.2 (44.2-54.1) |
| Lleida | 709 | 55.6 (52.0-59.1) | 336 | 59.5 (53.0-65.6) | 373 | 51.6 (47.0-56.2) |
| Lugo | 743 | 66.8 (62.8-70.7) | 353 | 76.0 (71.8-79.7) | 390 | 58.5 (50.3-66.1) |
| Madrid | 3358 | 49.5 (47.2-51.9) | 1595 | 58.0 (55.3-60.7) | 1763 | 42.0 (38.9-45.2) |
| Málaga | 1246 | 58.0 (54.6-61.4) | 592 | 66.4 (62.2-70.3) | 654 | 50.2 (45.2-55.1) |
| Murcia | 1392 | 58.9 (55.0-62.8) | 643 | 67.3 (61.4-72.7) | 749 | 50.7 (46.5-54.9) |
| Navarra | 1519 | 50.1 (46.6-53.7) | 750 | 59.4 (55.3-63.4) | 769 | 41.2 (36.4-46.2) |
| Ourense | 749 | 59.3 (55.1-63.4) | 335 | 68.1 (62.3-73.5) | 414 | 51.4 (45.8-56.9) |
| Palencia | 704 | 53.8 (48.0-59.6) | 335 | 61.3 (55.2-67.0) | 369 | 46.8 (40.3-53.5) |
| Palmas, Las | 1427 | 58.0 (54.0-61.9) | 655 | 63.1 (58.0-67.8) | 772 | 53.1 (47.7-58.5) |
| Pontevedra | 1177 | 60.5 (56.6-64.4) | 551 | 68.1 (64.1-71.8) | 626 | 53.7 (48.1-59.2) |
| Rioja, La | 1213 | 55.4 (52.8-58.0) | 592 | 63.7 (60.0-67.2) | 621 | 47.3 (43.4-51.3) |
| Salamanca | 751 | 55.0 (49.0-60.8) | 342 | 66.2 (59.6-72.2) | 409 | 44.7 (37.1-52.5) |
| Santa Cruz de Tenerife | 1229 | 62.5 (58.8-66.1) | 568 | 68.4 (63.6-72.8) | 661 | 56.8 (52.0-61.5) |
| Segovia | 643 | 53.3 (50.1-56.6) | 316 | 60.8 (56.3-65.1) | 327 | 46.1 (41.3-51.0) |
| Sevilla | 1531 | 58.5 (55.3-61.6) | 726 | 65.9 (61.8-69.7) | 805 | 51.7 (47.1-56.2) |
| Soria | 627 | 57.0 (49.9-63.8) | 315 | 62.6 (52.9-71.3) | 312 | 51.3 (44.7-57.8) |
| Tarragona | 870 | 58.7 (54.4-62.8) | 419 | 62.6 (57.9-67.0) | 451 | 54.8 (49.1-60.4) |
| Teruel | 594 | 57.1 (53.7-60.3) | 302 | 65.6 (60.6-70.3) | 292 | 48.4 (44.2-52.5) |
| Toledo | 1040 | 61.1 (56.4-65.6) | 503 | 69.8 (64.1-74.9) | 537 | 52.7 (46.6-58.7) |
| Valencia/València | 1834 | 54.1 (51.3-56.9) | 865 | 64.0 (60.8-67.0) | 969 | 44.7 (40.6-48.9) |
| Valladolid | 931 | 54.3 (50.1-58.5) | 435 | 62.9 (57.5-68.0) | 496 | 46.1 (40.1-52.3) |
| Zamora | 608 | 57.9 (52.6-62.9) | 300 | 65.6 (58.4-72.1) | 308 | 50.5 (43.5-57.4) |
| Zaragoza | 1170 | 55.1 (50.9-59.3) | 549 | 66.4 (62.0-70.5) | 621 | 44.5 (38.6-50.6) |
| Ceuta | 774 | 57.9 (52.8-62.8) | 346 | 63.3 (57.0-69.3) | 428 | 52.3 (45.6-58.9) |
| Melilla | 731 | 60.5 (56.2-64.6) | 330 | 65.2 (59.6-70.4) | 401 | 55.6 (49.8-61.3) |
