# Supplementary material for: Socio-geographical disparities of obesity and excess weight in adults in Spain: insights from the ENE-COVID study
Source: Front Public Health. 2023 Jul 17;11:1195249. doi: 10.3389/fpubh.2023.1195249 (PMC10387530; doi:10.3389/fpubh.2023.1195249)
Supplement: Supplementary file 9 [file Table_5.DOCX]

Supplementary Material

Socio-geographical disparities of obesity and excess of weight in adults in Spain: insights from the ENE-COVID study

**Enrique Gutiérrez-González, Marta García-Solano, Roberto Pastor-Barriuso, Nerea Fernández de Larrea-Baz, Almudena Rollán-Gordo, Belén Peñalver Argüeso, Isabel Peña-Rey^4^, Marina Pollán, Beatriz Pérez-Gómez and the ENE-COVID Study Group**

*** Correspondence:**Beatriz Pérez Gómez [bperez@isciii.es](mailto:bperez@isciii.es)

**Supplementary Table S5**: Age-standardized prevalence of obesity by sex and province in adults in ENE-COVID study

|  | **TOTAL** | | **MEN** | | **WOMEN** | |
| --- | --- | --- | --- | --- | --- | --- |
|  | **N** | **% (95% CI)** | **N** | **% (95% CI)** | **N** | **% (95% CI)** |
| Spain | 57131 | 18.7 (18.1-19.2) | 27031 | 19.6 (18.9-20.2) | 30100 | 17.8 (17.2-18.4) |
| **Province** |  |  |  |  |  |  |
| Albacete | 943 | 23.4 (18.9-28.6) | 459 | 22.0 (18.0-26.6) | 484 | 24.7 (20.6-29.2) |
| Alicante/Alacant | 1507 | 19.6 (17.2-22.2) | 714 | 21.7 (18.2-25.6) | 793 | 17.5 (14.1-21.5) |
| Almería | 844 | 20.8 (16.3-26.1) | 393 | 22.4 (16.1-30.3) | 451 | 19.2 (13.0-27.3) |
| Araba/Álava | 693 | 17.8 (14.6-21.6) | 333 | 17.6 (14.0-22.0) | 360 | 17.9 (14.3-22.2) |
| Asturias | 1604 | 20.0 (18.0-22.1) | 733 | 21.2 (18.5-24.2) | 871 | 18.6 (15.9-21.7) |
| Ávila | 619 | 19.5 (16.3-23.1) | 306 | 25.6 (20.8-31.1) | 313 | 13.6 (9.2-19.6) |
| Badajoz | 1415 | 27.0 (23.1-31.3) | 705 | 27.6 (23.0-32.7) | 710 | 26.1 (21.6-31.3) |
| Balears, Illes | 1214 | 16.6 (14.5-19.0) | 579 | 17.6 (14.8-20.8) | 635 | 15.6 (12.8-18.8) |
| Barcelona | 3307 | 16.8 (15.0-18.7) | 1536 | 17.3 (15.1-19.7) | 1771 | 16.1 (14.0-18.6) |
| Bizkaia | 1181 | 16.3 (12.7-20.7) | 550 | 16.4 (13.4-19.8) | 631 | 15.9 (13.0-19.4) |
| Burgos | 793 | 14.4 (12.1-17.1) | 391 | 18.0 (14.5-22.3) | 402 | 10.7 (7.4-15.2) |
| Cáceres | 1083 | 18.6 (15.7-21.9) | 528 | 20.6 (17.5-24.0) | 555 | 16.5 (13.5-20.0) |
| Cádiz | 1235 | 21.8 (19.0-25.0) | 572 | 20.5 (17.1-24.5) | 663 | 22.8 (19.3-26.8) |
| Cantabria | 1480 | 17.9 (14.8-21.5) | 713 | 18.4 (15.3-21.9) | 767 | 17.3 (14.2-20.8) |
| Castellón/Castelló | 754 | 20.2 (16.8-24.0) | 361 | 21.0 (16.8-25.9) | 393 | 19.3 (15.1-24.2) |
| Ciudad Real | 1034 | 20.3 (17.1-23.9) | 493 | 23.2 (18.8-28.3) | 541 | 17.4 (13.2-22.7) |
| Córdoba | 963 | 23.4 (19.6-27.8) | 444 | 23.4 (19.5-27.9) | 519 | 23.2 (19.2-27.7) |
| Coruña, A | 1193 | 21.5 (18.6-24.7) | 548 | 20.4 (15.3-26.6) | 645 | 22.1 (17.0-28.2) |
| Cuenca | 753 | 24.0 (20.1-28.3) | 382 | 25.7 (21.1-30.8) | 371 | 22.3 (17.9-27.5) |
| Gipuzkoa | 934 | 14.1 (10.7-18.4) | 450 | 14.9 (10.8-20.1) | 484 | 13.2 (9.3-18.6) |
| Girona | 1022 | 14.0 (11.4-17.1) | 457 | 13.4 (10.3-17.2) | 565 | 14.6 (11.5-18.4) |
| Granada | 945 | 22.2 (19.3-25.4) | 437 | 26.8 (21.8-32.3) | 508 | 17.8 (13.1-23.7) |
| Guadalajara | 742 | 16.7 (13.7-20.2) | 359 | 18.2 (13.8-23.7) | 383 | 15.2 (10.9-20.9) |
| Huelva | 855 | 21.6 (17.5-26.3) | 393 | 23.8 (17.4-31.6) | 462 | 19.2 (13.1-27.4) |
| Huesca | 659 | 15.4 (12.6-18.7) | 301 | 13.9 (10.4-18.3) | 358 | 16.7 (13.1-21.0) |
| Jaén | 921 | 22.0 (19.4-24.8) | 433 | 25.3 (21.4-29.7) | 488 | 18.6 (14.8-23.2) |
| León | 868 | 13.5 (11.1-16.2) | 408 | 13.4 (9.7-18.2) | 460 | 13.3 (9.6-18.1) |
| Lleida | 709 | 18.8 (15.2-23.2) | 336 | 18.8 (14.8-23.6) | 373 | 18.7 (14.7-23.4) |
| Lugo | 743 | 25.8 (20.7-31.5) | 353 | 25.2 (20.3-30.8) | 390 | 26.1 (21.2-31.7) |
| Madrid | 3358 | 14.9 (13.3-16.5) | 1595 | 16.6 (14.7-18.7) | 1763 | 13.2 (11.3-15.3) |
| Málaga | 1246 | 20.2 (16.6-24.4) | 592 | 20.4 (15.7-26.0) | 654 | 19.8 (15.2-25.5) |
| Murcia | 1392 | 19.7 (17.0-22.7) | 643 | 20.2 (16.4-24.5) | 749 | 19.2 (15.5-23.6) |
| Navarra | 1519 | 15.6 (13.0-18.7) | 750 | 16.3 (13.6-19.5) | 769 | 14.8 (12.1-18.0) |
| Ourense | 749 | 17.2 (14.5-20.4) | 335 | 20.6 (16.9-24.9) | 414 | 14.1 (10.6-18.6) |
| Palencia | 704 | 14.3 (10.5-19.2) | 335 | 17.0 (12.0-23.7) | 369 | 11.7 (7.0-18.9) |
| Palmas, Las | 1427 | 22.6 (19.5-26.1) | 655 | 21.7 (17.8-26.1) | 772 | 23.4 (19.5-27.8) |
| Pontevedra | 1177 | 21.6 (18.5-25.1) | 551 | 20.6 (16.9-24.9) | 626 | 22.2 (18.5-26.4) |
| Rioja, La | 1213 | 16.5 (14.1-19.2) | 592 | 17.2 (13.6-21.4) | 621 | 15.8 (12.2-20.1) |
| Salamanca | 751 | 13.9 (10.9-17.7) | 342 | 15.9 (11.8-21.1) | 409 | 12.1 (8.2-17.6) |
| Santa Cruz de Tenerife | 1229 | 23.4 (21.2-25.8) | 568 | 24.3 (20.7-28.4) | 661 | 22.5 (18.9-26.5) |
| Segovia | 643 | 16.7 (12.9-21.2) | 316 | 17.4 (13.4-22.3) | 327 | 15.8 (11.9-20.8) |
| Sevilla | 1531 | 23.5 (21.0-26.3) | 726 | 25.2 (21.5-29.3) | 805 | 21.8 (18.1-26.0) |
| Soria | 627 | 17.8 (14.4-21.7) | 315 | 19.6 (15.5-24.5) | 312 | 15.8 (11.8-20.9) |
| Tarragona | 870 | 22.0 (18.3-26.1) | 419 | 21.5 (17.9-25.7) | 451 | 22.4 (18.8-26.6) |
| Teruel | 594 | 22.4 (19.5-25.6) | 302 | 24.1 (18.6-30.7) | 292 | 20.7 (15.3-27.4) |
| Toledo | 1040 | 22.2 (18.6-26.3) | 503 | 22.1 (17.6-27.4) | 537 | 22.3 (17.7-27.5) |
| Valencia/València | 1834 | 17.6 (16.0-19.2) | 865 | 17.2 (14.6-20.3) | 969 | 17.7 (15.0-20.7) |
| Valladolid | 931 | 14.6 (11.7-18.1) | 435 | 14.5 (10.7-19.4) | 496 | 14.6 (10.8-19.4) |
| Zamora | 608 | 17.0 (12.6-22.4) | 300 | 17.6 (13.0-23.4) | 308 | 16.2 (11.6-22.1) |
| Zaragoza | 1170 | 18.3 (15.8-21.2) | 549 | 19.2 (16.4-22.4) | 621 | 17.2 (14.4-20.5) |
| Ceuta | 774 | 19.5 (16.1-23.3) | 346 | 16.3 (13.2-20.0) | 428 | 22.6 (19.4-26.2) |
| Melilla | 731 | 20.9 (18.1-24.0) | 330 | 19.5 (16.1-23.5) | 401 | 22.4 (18.9-26.3) |
