# Supplementary material for: Socio-geographical disparities of obesity and excess weight in adults in Spain: insights from the ENE-COVID study
Source: Front Public Health. 2023 Jul 17;11:1195249. doi: 10.3389/fpubh.2023.1195249 (PMC10387530; doi:10.3389/fpubh.2023.1195249)
Supplement: Supplementary file 10 [file Table_6.DOCX]

Supplementary Material

Socio-geographical disparities of obesity and excess of weight in adults in Spain: insights from the ENE-COVID study

**Enrique Gutiérrez-González, Marta García-Solano, Roberto Pastor-Barriuso, Nerea Fernández de Larrea-Baz, Almudena Rollán-Gordo, Belén Peñalver Argüeso, Isabel Peña-Rey^4^, Marina Pollán, Beatriz Pérez-Gómez and the ENE-COVID Study Group**

*** Correspondence:**Beatriz Pérez Gómez [bperez@isciii.es](mailto:bperez@isciii.es)

**Supplementary Table S6**: Age-standardized prevalence of excess weight by sex and province in adults in ENE-COVID study

|  | **TOTAL** | | **MEN** | | **WOMEN** | |
| --- | --- | --- | --- | --- | --- | --- |
|  | **N** | **% (95% CI)** | **N** | **% (95% CI)** | **N** | **% (95% CI)** |
| Spain | 57131 | 55.8 (55.1-56.4) | 27031 | 64.2 (63.4-64.9) | 30100 | 47.8 (46.9-48.7) |
| **Province** |  |  |  |  |  |  |
| Albacete | 943 | 61.6 (54.5-68.3) | 459 | 70.9 (65.6-75.6) | 484 | 52.7 (47.6-57.7) |
| Alicante/Alacant | 1507 | 55.5 (52.5-58.3) | 714 | 65.4 (61.3-69.2) | 793 | 46.1 (42.2-50.1) |
| Almería | 844 | 65.6 (59.8-71.0) | 393 | 71.9 (65.1-77.8) | 451 | 59.6 (53.1-65.8) |
| Araba/Álava | 693 | 55.7 (49.9-61.3) | 333 | 61.0 (53.4-68.2) | 360 | 50.3 (43.0-57.7) |
| Asturias | 1604 | 57.2 (54.4-59.9) | 733 | 66.1 (63.2-68.9) | 871 | 48.6 (45.7-51.4) |
| Ávila | 619 | 54.7 (51.1-58.2) | 306 | 65.2 (60.4-69.8) | 313 | 44.6 (39.9-49.3) |
| Badajoz | 1415 | 64.5 (62.2-66.8) | 705 | 70.7 (67.4-73.8) | 710 | 58.6 (55.4-61.8) |
| Balears, Illes | 1214 | 53.7 (50.5-56.9) | 579 | 63.1 (58.3-67.7) | 635 | 44.5 (39.9-49.3) |
| Bizkaia | 1181 | 51.4 (47.0-55.8) | 550 | 57.1 (52.6-61.4) | 631 | 45.7 (41.4-50.2) |
| Barcelona | 3307 | 53.4 (50.7-56.1) | 1536 | 61.2 (58.7-63.7) | 1771 | 45.7 (43.2-48.2) |
| Burgos | 793 | 53.7 (47.9-59.5) | 391 | 61.9 (56.8-66.7) | 402 | 45.9 (41.0-50.9) |
| Cáceres | 1083 | 56.4 (51.9-60.7) | 528 | 66.3 (62.1-70.2) | 555 | 46.8 (42.7-50.9) |
| Cádiz | 1235 | 61.3 (58.6-64.0) | 572 | 68.0 (64.4-71.4) | 663 | 54.9 (51.3-58.3) |
| Cantabria | 1480 | 54.1 (50.5-57.6) | 713 | 61.4 (57.0-65.7) | 767 | 46.9 (42.6-51.2) |
| Castellón/Castelló | 754 | 55.7 (51.8-59.6) | 361 | 63.8 (60.1-67.4) | 393 | 47.9 (44.3-51.6) |
| Ciudad Real | 1034 | 57.6 (53.7-61.4) | 493 | 65.7 (60.3-70.8) | 541 | 49.7 (44.5-54.9) |
| Córdoba | 963 | 63.9 (59.0-68.5) | 444 | 73.7 (69.2-77.7) | 519 | 54.4 (50.1-58.6) |
| Coruña, A | 1193 | 60.5 (56.4-64.4) | 548 | 65.8 (60.7-70.5) | 645 | 55.1 (50.1-59.9) |
| Cuenca | 753 | 56.9 (51.8-61.8) | 382 | 65.9 (59.7-71.7) | 371 | 48.1 (42.1-54.1) |
| Gipuzkoa | 934 | 49.7 (46.0-53.5) | 450 | 58.1 (53.8-62.3) | 484 | 41.6 (37.4-45.9) |
| Girona | 1022 | 48.1 (44.7-51.5) | 457 | 57.6 (54.0-61.1) | 565 | 39.0 (35.5-42.6) |
| Granada | 945 | 58.0 (53.5-62.4) | 437 | 67.6 (63.0-72.0) | 508 | 48.7 (44.2-53.2) |
| Guadalajara | 742 | 53.2 (48.9-57.4) | 359 | 61.9 (56.4-67.1) | 383 | 44.7 (39.4-50.1) |
| Huelva | 855 | 63.0 (58.6-67.3) | 393 | 71.6 (66.4-76.4) | 462 | 54.8 (49.8-59.8) |
| Huesca | 659 | 53.8 (48.8-58.7) | 301 | 64.7 (59.3-69.7) | 358 | 43.3 (38.1-48.6) |
| Jaén | 921 | 61.0 (56.7-65.2) | 433 | 66.4 (61.2-71.2) | 488 | 55.8 (50.7-60.8) |
| León | 868 | 56.2 (51.4-60.9) | 408 | 66.4 (60.7-71.7) | 460 | 46.5 (41.0-52.1) |
| Lleida | 709 | 54.8 (51.1-58.4) | 336 | 60.0 (53.7-66.0) | 373 | 49.9 (43.8-56.1) |
| Lugo | 743 | 64.0 (59.7-68.2) | 353 | 73.3 (68.4-77.7) | 390 | 55.1 (50.4-59.7) |
| Madrid | 3358 | 50.5 (48.1-52.9) | 1595 | 59.1 (56.5-61.7) | 1763 | 42.2 (39.6-44.8) |
| Málaga | 1246 | 58.3 (55.1-61.5) | 592 | 66.6 (62.6-70.4) | 654 | 50.2 (46.3-54.1) |
| Murcia | 1392 | 60.2 (56.5-63.8) | 643 | 69.0 (63.8-73.8) | 749 | 51.7 (46.6-56.7) |
| Navarra | 1519 | 49.6 (46.2-53.0) | 750 | 59.6 (55.8-63.3) | 769 | 40.1 (36.5-43.9) |
| Ourense | 749 | 55.8 (52.2-59.3) | 335 | 65.2 (59.4-70.6) | 414 | 46.8 (41.2-52.4) |
| Palencia | 704 | 51.4 (45.3-57.5) | 335 | 59.2 (52.8-65.3) | 369 | 43.9 (37.8-50.3) |
| Palmas, Las | 1427 | 58.8 (54.9-62.7) | 655 | 64.3 (59.9-68.5) | 772 | 53.5 (49.3-57.8) |
| Pontevedra | 1177 | 59.5 (55.6-63.3) | 551 | 67.4 (63.3-71.1) | 626 | 51.8 (47.9-55.7) |
| Rioja, La | 1213 | 54.1 (51.5-56.6) | 592 | 63.0 (59.5-66.4) | 621 | 45.5 (42.1-48.9) |
| Salamanca | 751 | 52.8 (47.4-58.2) | 342 | 64.7 (58.8-70.2) | 409 | 41.5 (35.9-47.4) |
| Santa Cruz de Tenerife | 1229 | 63.4 (59.6-67.0) | 568 | 69.6 (64.5-74.2) | 661 | 57.4 (52.5-62.2) |
| Segovia | 643 | 51.9 (49.1-54.7) | 316 | 59.8 (55.4-64.0) | 327 | 44.4 (40.2-48.7) |
| Sevilla | 1531 | 59.3 (56.4-62.2) | 726 | 66.8 (63.3-70.1) | 805 | 52.1 (48.6-55.5) |
| Soria | 627 | 54.8 (48.9-60.6) | 315 | 61.7 (53.3-69.4) | 312 | 48.3 (40.3-56.4) |
| Tarragona | 870 | 58.3 (54.4-62.1) | 419 | 62.8 (58.7-66.7) | 451 | 54.2 (50.2-58.2) |
| Teruel | 594 | 55.5 (52.1-58.9) | 302 | 64.9 (59.5-69.9) | 292 | 46.4 (41.3-51.7) |
| Toledo | 1040 | 61.1 (56.7-65.4) | 503 | 70.1 (65.2-74.7) | 537 | 52.4 (47.6-57.1) |
| Valencia/València | 1834 | 54.2 (51.5-56.8) | 865 | 64.8 (61.7-67.9) | 969 | 43.9 (40.9-47.0) |
| Valladolid | 931 | 52.5 (48.8-56.1) | 435 | 61.6 (56.8-66.2) | 496 | 43.9 (39.2-48.6) |
| Zamora | 608 | 54.1 (47.9-60.1) | 300 | 62.3 (54.8-69.3) | 308 | 46.1 (38.9-53.4) |
| Zaragoza | 1170 | 54.1 (50.2-57.9) | 549 | 66.3 (62.3-70.1) | 621 | 42.4 (38.6-46.4) |
| Ceuta | 774 | 60.5 (55.3-65.6) | 346 | 66.2 (60.0-71.9) | 428 | 55.1 (49.1-61.0) |
| Melilla | 731 | 63.1 (58.8-67.2) | 330 | 68.3 (63.0-73.1) | 401 | 58.4 (53.3-63.3) |
